# TAO-DFT fictitious temperature made simple†

**DOI:** 10.1039/d2ra01632j

**Published:** 2022-04-22

**Authors:** Bo-Jyun Chen, Jeng-Da Chai

**Affiliations:** Department of Physics, National Taiwan University Taipei 10617 Taiwan jdchai@phys.ntu.edu.tw; Center for Theoretical Physics, Center for Quantum Science and Engineering, National Taiwan University Taipei 10617 Taiwan; Physics Division, National Center for Theoretical Sciences Taipei 10617 Taiwan

## Abstract

Over the past few years, thermally-assisted-occupation density functional theory (TAO-DFT) [J.-D. Chai, *J. Chem. Phys.*, 2012, **136**, 154104] has been proved to be an efficient electronic structure method for investigating the ground-state properties of large electronic systems with strong static correlation effects. In TAO-DFT, the strength of static correlation in an electronic system at zero temperature is closely related to the so-called fictitious temperature (*i.e.*, the temperature of the corresponding noninteracting reference system). In this work, we propose a simple model to define the optimal system-independent fictitious temperature of a given energy functional in TAO-DFT. Besides, we employ this model to determine the optimal system-independent fictitious temperature of a global hybrid functional in TAO-DFT as a function of the fraction of exact exchange. In addition, we adopt TAO-DFT with various global hybrid functionals and system-independent fictitious temperatures to explore the ground-state properties of several electronic systems with strong static correlation effects, such as the linear acenes and cyclic carbon chains. Furthermore, we discuss the role of exact exchange and an optimal system-independent fictitious temperature in TAO-DFT. Owing to the much reduced self-interaction error, TAO-DFT with exact exchange and an optimal system-independent fictitious temperature can accurately predict the radical character and bond length alternation of cyclic carbon chains (with even number of carbon atoms), which are challenging problems for traditional electronic structure methods.

## Abstract 2

Optimal system-independent fictitious temperature *θ* of TAO-GH as a function of the fraction of exact exchange *a*_x_.

## Graphical abstract

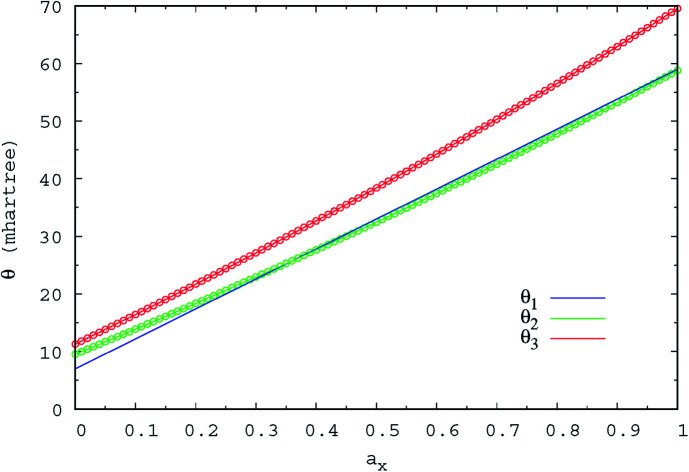

## I. Introduction

Over the past three decades, Kohn–Sham density functional theory (KS-DFT)^1,2^ has been a major workhorse for electronic structure calculations in condensed matter physics, quantum chemistry, and materials science, because of its low computational cost and reasonable accuracy for the ground-state properties of a wide range of electronic systems. Nonetheless, in KS-DFT, the exact exchange–correlation (XC) energy functional *E*_xc_[*ρ*] remains unknown, and hence, density functional approximations (DFAs) for *E*_xc_[*ρ*] are needed for practical electronic structure calculations.^3–6^

In KS-DFT, the XC energy functionals based on the commonly used DFAs (*e.g.*, the local density approximation (LDA)^7,8^ and generalized gradient approximations (GGAs)^9–11^) are very promising in terms of computational efficiency. Nevertheless, the DFA XC energy functionals can possess a number of inherent drawbacks,^3–6,12,13^ which are commonly categorized into three qualitative errors: the self-interaction error (SIE), non-covalent interaction error (NCIE), and static correlation error (SCE). In general, these errors can be greatly reduced by making some modifications to the parent DFA XC energy functionals: the mixing of Hartree–Fock (HF) exchange energy (*i.e.*, the so-called hybrid functionals^14–24^) for the SIE; the introduction of dispersion correction^25,26^ or second-order Møller–Plesset (MP2) correlation energy (*i.e.*, the so-called double-hybrid (DH) functionals,^17,27^ wherein the mixing of HF exchange energy is also performed) for the NCIE; the inclusion of fully nonlocal correlation energy, such as the random phase approximation (RPA) correlation energy,^28,29^ for the SCE. Note, however, that in KS-DFT, the popular hybrid and DH functionals fail to describe static correlation, and the RPA and related functionals can be computationally very demanding for large electronic systems.

Aiming to describe static correlation with low computational complexity (*i.e.*, for the ground-state properties of large electronic systems with strong static correlation effects), thermally-assisted-occupation density functional theory (TAO-DFT)^30–32^ has been recently developed. Unlike KS-DFT, the ground-state density of an electronic system in TAO-DFT is represented with the thermally-assisted-occupation (TAO) orbitals and their occupation numbers. The TAO orbital occupation numbers (TOONs) are given by the Fermi–Dirac (FD) distribution function with a fictitious temperature *θ*, *i.e.*, the temperature of the corresponding noninteracting reference system in TAO-DFT. Note that TAO-DFT (with *θ* = 0) is reduced to KS-DFT. In strong contrast to finite-temperature density functional theory (FT-DFT) (first proposed by Mermin^33^ and subsequently powered by the Mermin–Kohn–Sham equation^2^ for practical calculations), which was developed for the thermal equilibrium properties of electronic systems at finite electronic temperatures, TAO-DFT is a density functional theory for the ground-state properties of electronic systems at zero electronic temperature (just like KS-DFT). In other words, for the ground-state properties of electronic systems at zero electronic temperature, FT-DFT is identical to KS-DFT, while TAO-DFT (with *θ* ≠ 0) can be distinctly different from KS-DFT (especially for electronic systems with strong static correlation effects).

The commonly used DFA^30,31^ and global hybrid (GH)^32^ XC energy functionals (as defined in KS-DFT) can also be adopted in TAO-DFT, with the introduction and the subsequent approximations of *θ*-dependent energy functionals, such as *E*_*θ*_[*ρ*] (*i.e.*, the difference between the noninteracting kinetic free energy at zero temperature and that at the fictitious temperature *θ*; *e.g.*, see eqn (14) of ref. 30) and *E*_x,*θ*_[*ρ*] (*i.e.*, the difference between the exchange free energy at zero temperature and that at the fictitious temperature *θ*; *e.g.*, see eqn (25) of ref. 32). In principle, the range-separated hybrid (RSH) XC energy functionals^32,34^ can also be employed in TAO-DFT, once the corresponding *θ*-dependent energy functionals are developed. Note that at *θ* = 0, since the *θ*-dependent energy functionals vanish, TAO-DFT with a given energy functional (*i.e.*, a combined XC and *θ*-dependent energy functional)^30–32^ is reduced to KS-DFT with the corresponding XC energy functional.

Very recently, TAO-DFT has been extended to study the excited-state properties of electronic systems.^35^ Besides, TAO-DFT has also been combined with *ab initio* molecular dynamics (AIMD) to explore the dynamical properties of large electronic systems with strong static correlation effects.^36^ Moreover, TAO-DFT has been successfully employed to study the properties of large electronic systems with strong static correlation effects.^37–49^

In TAO-DFT, the fictitious temperature *θ* is a key ingredient. As discussed in our previous work,^30–32^ for an electronic system, the fictitious temperature *θ* of a given energy functional in TAO-DFT should be so chosen that the distribution of TOONs is close to the distribution of the exact natural orbital occupation numbers (NOONs),^50^ which is closely related to the stability (*i.e.*, the single-reference (SR)/multi-reference (MR) character) of an electronic system. In such a situation, the static correlation of an electronic system can be adequately described by the entropy contribution (*e.g.*, see eqn (26) and Section III.E of ref. 30) in TAO-DFT. In other words, the optimal *θ* value should be closely related to the SR/MR character of an electronic system. Accordingly, a self-consistent scheme for defining the optimal *θ* values of electronic systems has been recently proposed.^51^ However, the system-dependent *θ* scheme can be computationally expensive for studying large electronic systems (especially for geometry optimizations wherein it is necessary to frequently update the optimal *θ* values).

Owing to the aforementioned reasons, for a given energy functional in TAO-DFT, although it is unlikely to employ a single value of *θ* (*i.e.*, a system-independent *θ*) that can be optimal for all electronic systems, TAO-DFT with such a system-independent *θ* scheme^30–32^ is as computationally efficient as KS-DFT, and can be reasonably accurate for a wide range of SR and MR systems, providing that the system-independent *θ* can be properly defined. In our previous work,^30–32^ for a given energy functional in TAO-DFT, the optimal system-independent *θ* has been defined as the largest *θ* with which TAO-DFT employing the energy functional can perform comparably to TAO-DFT employing the same energy functional at *θ* = 0 (*i.e.*, KS-DFT employing the corresponding XC energy functional) for SR systems. In other words, for SR systems, the results obtained from TAO-DFT with this *θ* can only yield acceptable deviations from those obtained from the KS-DFT counterpart. It has been shown that TAO-DFT with this choice of *θ*, which is as computationally efficient as KS-DFT, can perform comparably to KS-DFT for several SR systems,^30–32,51^ and can outperform KS-DFT for various MR systems.^30–32,37–49,51^

However, in the previous work,^30–32^ the optimal system-independent *θ* values of energy functionals (*e.g.*, the LDA, GGA, and GH functionals) in TAO-DFT have been determined based on their performance (*i.e.*, relative to the KS-DFT counterpart) on some training set (containing the energetic and geometric properties of several SR systems). It is necessary to perform several TAO-DFT calculations with a wide range of *θ* values on the training set to approximately locate the optimal *θ* values. Besides, the selection of optimal *θ* values can be tricky, as it remains ambiguous to judge the extent to which the deviations are acceptable. To overcome these issues, in the present work, we propose a simple model to define the optimal system-independent *θ* value of a given energy functional in TAO-DFT.

The rest of this paper is organized as follows. In Section II, we describe this simple model. In Section III, we employ this model to determine the optimal system-independent *θ* values of GH functionals (*i.e.*, including the DFA functionals) in TAO-DFT. In Section IV, we examine the performance of TAO-DFT with various GH functionals and system-independent *θ* values on the ground-state properties of several electronic systems with strong static correlation effects, such as the linear acenes and cyclic carbon chains. Our conclusions are given in Section V.

## II. Simple model for the optimal system-independent fictitious temperature in TAO-DFT

Consider a singlet ground-state system of *N* electrons moving in the presence of an external potential *v*_ext_(**r**) at zero electronic temperature, wherein the standard computational approach, *i.e.*, the spin-restricted (spin-unpolarized) formalism, is adopted. If the singlet ground-state system is a perfect closed-shell system, all the NOONs must be either 0 (fully empty) or 2 (fully occupied), and hence the optimal *θ* value in TAO-DFT must be strictly zero,^30–32^ wherein TAO-DFT (with *θ* = 0) is reduced to KS-DFT. However, among real electronic systems, such a perfect closed-shell system, which possesses perfect nonradical character, can hardly be found.^52^

If the singlet ground-state system possesses strong nonradical character (*i.e.*, a typical SR system), all the NOONs should remain in the vicinity of either 0 or 2, and hence the optimal *θ* value in TAO-DFT should remain sufficiently small. Therefore, for such an electronic system, the energy gap between the (*N*/2)-th orbital (*i.e.*, the highest occupied molecular orbital (HOMO)) and the (*N*/2 + 1)-th orbital (*i.e.*, the lowest unoccupied molecular orbital (LUMO)), *i.e.*, the HOMO–LUMO (HL) gap in TAO-DFT, which should be very close to the HL gap in KS-DFT (*i.e.*, TAO-DFT with *θ* = 0), is expected to be very large.^53^

For the NOONs of most SR systems, it is adequate to retain only the highest occupied natural orbital (HONO) occupation number *n*_HONO_, and to approximate the lowest unoccupied natural orbital (LUNO) occupation number by (2 − *n*_HONO_) and all the other NOONs by either 0 or 2. Accordingly, the NOONs of most SR systems can be approximately expressed as1
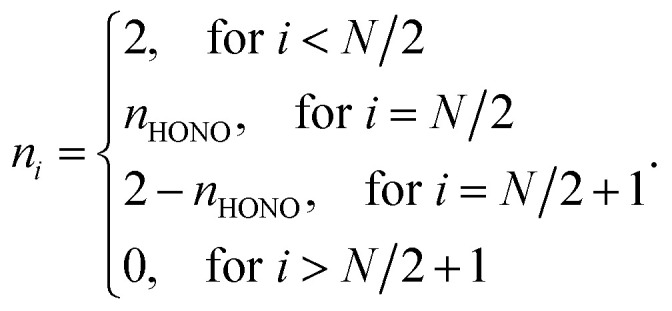


For most SR systems, *n*_HONO_ should be slightly less than 2. On the other hand, the NOONs of MR systems possessing diradical character can also be described by eqn (1), wherein the HONO occupation number should be close to 1. Therefore, it is reasonable to assume that the *n*_HONO_ values of most SR systems should obey

2 *n*_HONO_ ≳ *n*_0_ ≫ 1

where *n*_0_ is the HONO occupation number of an RES (reference electronic system) possessing slightly weaker nonradical character than most SR systems, and much weaker diradical character than MR systems with diradical character. The RES, which possesses moderate nonradical character (together with only a low degree of diradical character), can be approximately regarded as a boundary between most SR systems and MR systems with diradical character. As the RES should possess slightly weaker nonradical character than most SR systems, in TAO-DFT, the optimal *θ* value of RES should be slightly larger than the optimal *θ* values of most SR systems.^30–32^ Therefore, in the present work, we define the optimal system-independent *θ* value of a given energy functional in TAO-DFT as the corresponding optimal *θ* value of RES, which can approximately meet the criterion of optimal system-independent *θ* defined in our previous work^30–32^ (as will be shown later).

In TAO-DFT,^30–32^ for a given fictitious temperature *θ*, the ground-state electron density *ρ*(**r**) of RES can be represented with the TAO orbitals {*ψ*_*i*_(**r**)} and their occupation numbers (*i.e.*, TOONs) {*f*_*i*_} (atomic units are adopted throughout this work):3
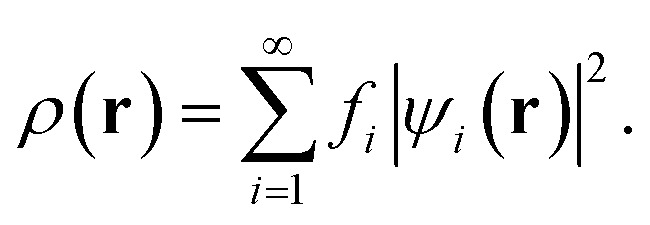
Here, *f*_*i*_ is the occupation number of the *i*-th TAO orbital *ψ*_*i*_(**r**), given by the FD distribution function (which has been multiplied by 2 due to spin degeneracy):

4 *f*_*i*_ = 2{1 + exp[(*ε*_*i*_ − *μ*)/*θ*]}^−1^

obeying the conditions: 
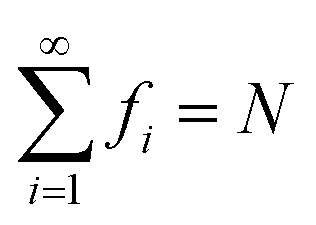
 and 0 ≤ *f*_*i*_ ≤ 2, where *ε*_*i*_ is the energy of the *i*-th TAO orbital *ψ*_*i*_(**r**), and *μ* is the chemical potential chosen for the conservation of the number of electrons *N*. From eqn (4), we obtain5
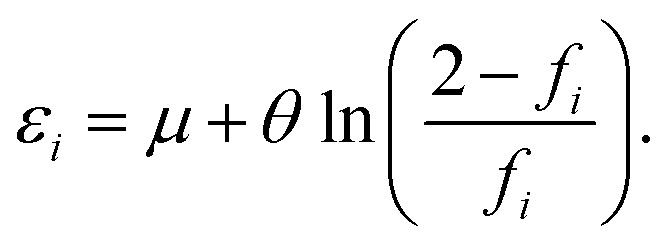


Therefore, the HL gap of RES obtained from TAO-DFT can be expressed as6
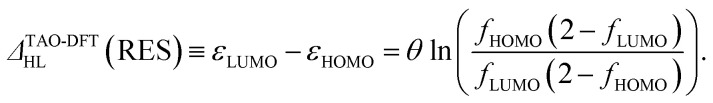


Hence, the fictitious temperature *θ* can be expressed as7
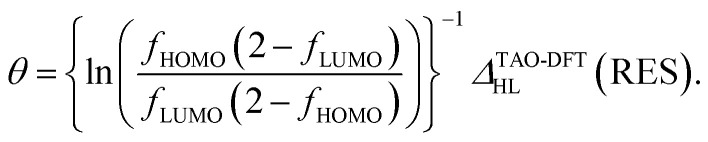


At the optimal *θ* value of RES, since the TOONs should be good approximations of the exact or reliably accurate NOONs,^30–32^*f*_HOMO_ and *f*_LUMO_ can naturally be replaced with the HONO occupation number (*n*_0_) and the LUNO occupation number (≈2 − *n*_0_), respectively, of RES, obtained from the exact theory or a reliably accurate MR electronic structure method:

8 *f*_HOMO_ ≈ *n*_0_, *f*_LUMO_ ≈ 2 − *n*_0_.

Moreover, since the RES possesses moderate nonradical character (together with only a low degree of diradical character), the HL gap of RES obtained from TAO-DFT (with the optimal *θ* value of RES) can be approximated by the HL gap of RES obtained from TAO-DFT (with *θ* = 0), *i.e.*, the HL gap of RES obtained from the KS-DFT counterpart *Δ*^KS-DFT^_HL_(RES):

9 *Δ*^TAO-DFT^_HL_(RES) ≈ *Δ*^KS-DFT^_HL_(RES).

Applying eqn (8) and (9) to eqn (7), the optimal *θ* value of RES can be approximately expressed as10
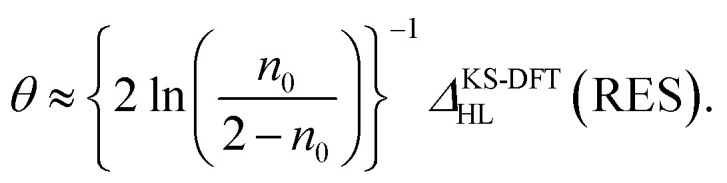


Eqn (10) is a key result in the present work, showing that the optimal system-independent *θ* value of a given energy functional in TAO-DFT, which is defined as the corresponding optimal *θ* value of RES, can be approximately expressed as a function of *n*_0_ (*i.e.*, the HONO occupation number of RES obtained from the exact theory or a reliably accurate MR electronic structure method) and *Δ*^KS-DFT^_HL_(RES) (*i.e.*, the HL gap of RES obtained from the KS-DFT counterpart). Since the latter can be dependent on the choice of XC energy functionals, the optimal *θ* value of RES (given by eqn (10)) is naturally dependent on the XC energy functional adopted (*e.g.*, the LDA, GGA, and GH functionals), *i.e.*, the energy functional adopted in TAO-DFT (with *θ* = 0).

Here, we discuss the choice of RES. Selecting a proper RES can be a nontrivial task, as the boundary between most SR systems and MR systems with diradical character may appear blurry. To properly select an RES which can best catch the essence of the nonradical/diradical boundary, some guidelines are listed below. First, the simpler one is the better, just like that the uniform electron gas (UEG) has been chosen as the model system when devising the LDA XC^7,8^ and *θ*-dependent^30,32^ energy functionals. Second, the RES should not be too unstable to synthesize by conventional methods, and there should be sufficient experimental and theoretical evidence supporting that the RES can be approximately regarded as a boundary between most SR systems and MR systems with diradical character. Last but not least, it is desirable that the RES belongs to a series of electronic systems showing a clear transition from nonradical character to diradical character with increasing system size, from which an intermediate electronic system can be easily identified as the RES.

Now, we start searching for such an RES. The helium atom, which appears to be a simple electronic system, is inappropriate for an RES, as it possesses strong nonradical character. The dissociation of H_2_ can provide a transition from nonradical character (*i.e.*, near the equilibrium geometry) to diradical character (*i.e.*, at the dissociation limit) with increasing internuclear separation. However, it remains difficult to define the extent of bond stretching that corresponds to the nonradical/diradical boundary. The Coulson–Fischer (CF) point (*i.e.*, the point beyond which the spin-unrestricted and spin-restricted solutions of a SR electronic structure method (*e.g.*, the HF method and KS-DFT with the conventional LDA, GGA, and GH functionals) begin to differ due to spin-symmetry breaking) may seem to mark the point of such a nonradical/diradical transition.^54^ However, as this point does not exist for the exact theory or a reliably accurate MR electronic structure method, which does not suffer from the spin-symmetry breaking issue, it can be understood that the CF point is simply a sign showing where a SR electronic structure method reaches its limitation, rather than an essential turning point of the system where it undergoes an immediate transition from nonradical character to diradical character. Therefore, despite its simplicity, the stretched H_2_ at the CF point is also inappropriate for an RES.

Perhaps, polycyclic aromatic hydrocarbons (PAHs) are sensible and practical options, as a number of PAHs have been studied both experimentally and theoretically in recent years. At this stage, the question is how to fuse the fewest aromatic rings to achieve moderate diradical character such that the results from reliably accurate MR electronic structure methods can be available (due to their high computational expense for large electronic systems). A recent study^55^ has investigated the radical character of various PAHs with 4, 5, and 6 aromatic rings, and it has been shown that linear acenes (*i.e.*, *n*-acenes, consisting of *n* linearly fused benzene rings) generally possess stronger diradical character than the PAHs with the same number of fused benzene rings. Similar conclusions have also been drawn by another study,^56^ showing that *n*-acenes generally have the smallest HL gaps among the PAHs of comparable size. More importantly, relative to other PAHs, there are plenty of experimental and theoretical results for *n*-acenes.^30–32,53,55–89^ Therefore, our search for an RES is now restricted to *n*-acenes.

The electronic properties of *n*-acene are highly dependent on the acene length. For example, the reactivity of *n*-acene, which originates from its radical character, increases as *n* increases. Naturally, the next question is which *n*-acene should be chosen as the RES. From the theoretical point of view, there has long been discussion about when the radical character of *n*-acene starts to emerge as *n* increases. The smaller *n*-acenes (*e.g.*, *n* ≤ 4) are known to possess strong nonradical character, indicating that they should belong to SR systems. On the other hand, 5-acene (*i.e.*, pentacene) has been widely regarded as an intermediate in radical character, just as it has served as a criterion when comparing the number of effectively unpaired electrons associated with different electronic systems.^76^ In a study of reduced HL gaps for various PAHs,^53^ it has been shown that 5-acene is on the boundary of kinetically stable PAHs and chemically reactive PAHs. A recent research using ACI-DSRG-MRPT2, which is a reliably accurate MR electronic structure method combining the adaptive configuration interaction (ACI)^90^ with a density-fitted implementation of the second-order perturbative MR-driven similarity renormalization group (DSRG-MRPT2),^91^ has reported that the diradical character of *n*-acene should emerge from *n* = 6,^86^ supporting that 5-acene should be on the nonradical/diradical boundary. Furthermore, another recent study has reported that 5-acene is generally a molecule with nonradical character, while its diradical character can emerge when the molecular structure undergoes a slight fluctuation (*e.g.*, a thermal fluctuation of room-temperature level),^81^ further supporting that 5-acene should indeed be on the nonradical/diradical boundary.

From the experimental point of view, owing to their highly reactive nature, the experimental singlet–triplet gaps of *n*-acenes are only available up to 5-acene.^57–60^ Note that 5-acene has a comparable singlet–triplet gap as some typical diradicals, while it is stable enough to be thoroughly investigated. The balanced radical character and stability of 5-acene allow applications, such as a good hole-transporting semiconductor.^56,78,88,89^ By contrast, 6-acene (*i.e.*, hexacene) is too unstable to isolate, since it is very susceptible to light or air, and tends to dimerize even in dilute solutions.^66,74,79,82^ One approach to stabilize 6-acene is to add some manipulating substitution groups,^87^ while this would certainly alter the properties of 6-acene. Also, it has been reported that 6-acene with relatively bulky substituents can still dimerize even in the dark.^70^ Therefore, 6-acene and the larger *n*-acenes (which generally possess stronger radical character than 6-acene) should be inappropriate for the RES. As a result, 5-acene should be on the nonradical/diradical boundary from the experimental point of view as well.

**Fig. 1:**
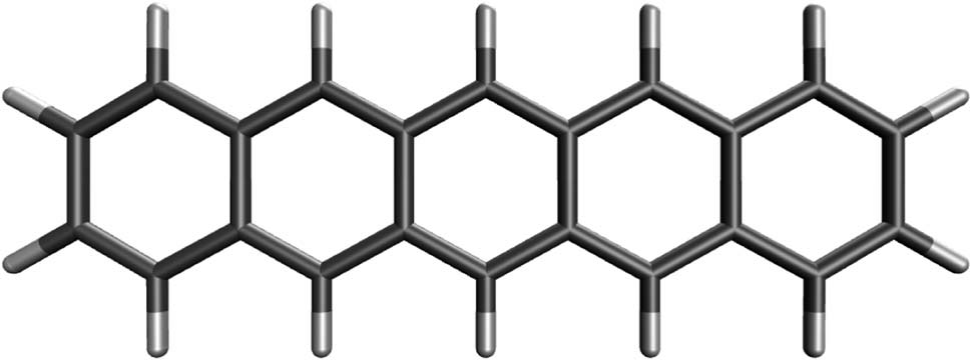
Reference electronic system (RES), 5-acene (*i.e.*, pentacene), containing five linearly fused benzene rings.

On the basis of all the theoretical and experimental reasons narrated above, it is sufficient to conclude that 5-acene (see Fig. 1) is ideal to serve as an RES. Accordingly, in this work, we choose 5-acene as the RES, due to the availability of reliably accurate results obtained from the recent experiments and MR electronic structure methods. For the HONO occupation number of 5-acene, we take *n*_0_ ≈ 1.8 recently obtained from a reliably accurate coupled-cluster valence-bond singles and doubles (CCVB-SD) calculation (with all valence electrons being correlated),^85^ and *n*_0_ = 1.73 obtained from a reliably accurate density matrix renormalization group (DMRG) calculation (using the DZ basis set).^64^ According to eqn (10), the optimal system-independent *θ* value of an energy functional (*i.e.*, a combined XC and *θ*-dependent energy functional) in TAO-DFT can be defined, respectively, by the *θ*_A_ model (with *n*_0_ ≈ 1.8):11
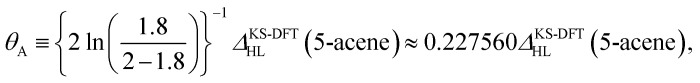
and by the *θ*_B_ model (with *n*_0_ = 1.73):12
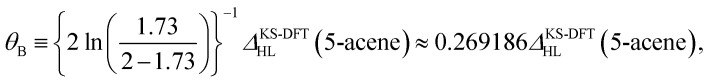
where *Δ*^KS-DFT^_HL_(5-acene) is the HL gap of 5-acene obtained from TAO-DFT with the energy functional at *θ* = 0, *i.e.*, KS-DFT with the corresponding XC energy functional.

## III. Optimal system-independent *θ* of a GH functional in TAO-DFT

A GH functional in TAO-DFT (denoted as TAO-GH)^32^ can be generally expressed as

13 *E*^TAO-GH^_xc_ = *a*_x_{*F*^HF,*θ*^_x_[{*f*_*i*_,*ψ*_*i*_}] + *E*^DFA^_x,*θ*_[*ρ*]} + (1 − *a*_x_)*E*^DFA^_x_[*ρ*] + *E*^DFA^_c_[*ρ*],

where *F*^HF,*θ*^_x_[{*f*_*i*_,*ψ*_*i*_}] is the HF exchange free energy of the TAO orbitals {*ψ*_*i*_(**r**)} and their occupation numbers {*f*_*i*_} at the fictitious temperature *θ* (*i.e.*, the exact exchange defined in TAO-DFT), *E*^DFA^_x_[*ρ*] is the DFA exchange energy, *E*^DFA^_c_[*ρ*] is the DFA correlation energy, *E*^DFA^_x,*θ*_[*ρ*] is the DFA for *E*_x,*θ*_[*ρ*], and *a*_x_ (*i.e.*, a value between 0 and 1) is the fraction of exact exchange. The corresponding ground-state energy in TAO-DFT is given by14
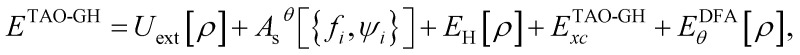
where the first term *U*_ext_[*ρ*] = ∫*ρ*(**r**)*v*_ext_(**r**)d**r** is the external potential energy, the second term *A*_s_^*θ*^[{*f*_*i*_,*ψ*_*i*_}] is the noninteracting kinetic free energy of the TAO orbitals {*ψ*_*i*_(**r**)} and their occupation numbers {*f*_*i*_} at the fictitious temperature *θ*, the third term 
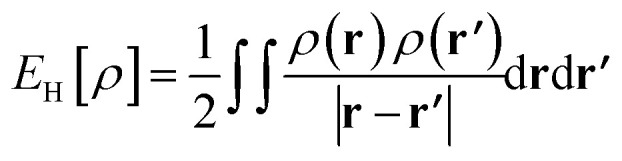
 is the Hartree energy, the fourth term *E*^TAO-GH^_xc_ is given by eqn (13), and the last term *E*^DFA^_*θ*_[*ρ*] is the DFA for *E*_*θ*_[*ρ*].

As discussed in our previous work,^30–32^ the choice of DFA functionals (*e.g.*, *E*^DFA^_x_[*ρ*], *E*^DFA^_c_[*ρ*], *E*^DFA^_*θ*_[*ρ*], and *E*^DFA^_x,*θ*_[*ρ*]) in TAO-DFT has insignificant effects on the optimal *θ* values, due to the similar TAO-orbital energy gaps. Therefore, the optimal *θ* value of TAO-GH is mainly dependent on the fraction of exact exchange *a*_x_. TAO-GH with a larger value of *a*_x_ yields larger TAO-orbital energy gaps, requiring a larger value of *θ* to yield a similar TOON distribution. In our previous work,^32^ the optimal system-independent *θ*, denoted as *θ*_1_ (in mhartree) in this work,

15 *θ*_1_ = 7 + 52*a*_x_

of TAO-GH, has been expressed as a linear function of *a*_x_. For the *a*_x_ = 0 case, *θ*_1_ = 7 mhartree, is correctly reduced to the optimal system-independent *θ* of TAO-DFA (*i.e.*, the DFA functional in TAO-DFT).^30,31^

In the present work, we adopt the *θ*_A_ (see eqn (11)) and *θ*_B_ (see eqn (12)) models to determine the optimal system-independent *θ* value of TAO-GH as a function of the fraction of exact exchange *a*_x_. For a given *a*_x_ (between 0 and 1), as the choice of DFA functionals in TAO-DFT has insignificant effects on the optimal *θ* values, we adopt the LDA, which is the simplest DFA, for all the DFA functionals in TAO-GH (see eqn (13) and (14)), *i.e.*, *E*^LDA^_x_[*ρ*],^7^*E*^LDA^_c_[*ρ*],^8^*E*^LDA^_*θ*_[*ρ*],^30^ and *E*^LDA^_x,*θ*_[*ρ*].^32^ The resulting TAO-GH is denoted as TAO-LDAh, which is given by

16 *E*^TAO-LDAh^_xc_ = *a*_x_{*F*^HF,*θ*^_x_[{*f*_*i*_,*ψ*_*i*_}] + *E*^LDA^_x,*θ*_[*ρ*]} + (1 − *a*_x_)*E*^LDA^_x_[*ρ*] + *E*^LDA^_c_[*ρ*],

and the corresponding ground-state energy in TAO-DFT is given by

17 *E*^TAO-LDAh^ = *U*_ext_[*ρ*] + *A*^*θ*^_s_[{*f*_*i*_,*ψ*_*i*_}] + *E*_H_[*ρ*] + *E*^TAO-LDAh^_xc_ + *E*^LDA^_*θ*_[*ρ*].

At *θ* = 0, as *E*^LDA^_*θ*=0_[*ρ*] = 0 and *E*^LDA^_x,*θ*=0_[*ρ*] = 0, TAO-LDAh reduces to KS-LDAh,^14,15^*i.e.*, KS-DFT with the corresponding XC energy functional:

18 *E*^KS-LDAh^_xc_ = *a*_x_*E*^HF^_x_[{*ϕ*_*i*_}] + (1 − *a*_x_)*E*^LDA^_x_[*ρ*] + *E*^LDA^_c_[*ρ*],

where *E*^HF^_x_[{*ϕ*_*i*_}] is the HF exchange energy of the occupied Kohn–Sham (KS) orbitals {*ϕ*_*i*_(**r**)}. The corresponding ground-state energy in KS-DFT is given by

19 *E*^KS-LDAh^ = *U*_ext_[*ρ*] + *T*_s_[{*ϕ*_*i*_}] + *E*_H_[*ρ*] + *E*^KS-LDAh^_xc_,

where *T*_s_[{*ϕ*_*i*_}] is the noninteracting kinetic energy of the occupied KS orbitals {*ϕ*_*i*_(**r**)}.

On the other hand, TAO-LDAh with *a*_x_ = 0 reduces to TAO-LDA (*i.e.*, TAO-DFT with the LDA XC and *θ*-dependent functional), and KS-LDAh with *a*_x_ = 0 reduces to KS-LDA (*i.e.*, KS-DFT with the LDA XC functional).

In this work, all calculations are performed with a development version of Q-Chem 4.3.^92^ Results are computed using the 6-31G(d) basis set with the fine grid EML(75, 302), consisting of 75 Euler–Maclaurin radial grid points and 302 Lebedev angular grid points, unless noted otherwise.

To obtain the optimal system-independent *θ* of TAO-LDAh with a given value of *a*_x_ (0.00, 0.01, 0.02, …, 0.98, 0.99, and 1.00), which is defined as the corresponding optimal *θ* value of 5-acene (*i.e.*, the RES chosen in this work), we perform spin-restricted calculations using KS-LDAh (*i.e.*, TAO-LDAh with *θ* = 0) with the same *a*_x_ to obtain the HL gap (*i.e.*, *Δ*^KS-DFT^_HL_(5-acene)) for the lowest singlet state (*i.e.*, the ground state) of 5-acene on the respective geometry fully optimized at the same level of theory.

With the numerical values of *Δ*^KS-DFT^_HL_(5-acene) for all the *a*_x_ values examined (0.00, 0.01, 0.02, …, 0.98, 0.99, and 1.00), we obtain the numerical value of the optimal system-independent *θ* of TAO-LDAh as a function of the fraction of exact exchange *a*_x_. The numerical data, given by the *θ*_A_ model (see eqn (11)), can fit extremely well to the following [1/1] Padé approximant (*i.e.*, the deviation remains very small (within 0.01 mhartree) for each *a*_x_), denoted as *θ*_2_ (in mhartree):20
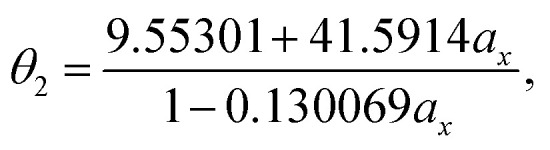
while the numerical data, given by the *θ*_B_ model (see eqn (12)), are found to fit extremely well to the following [1/1] Padé approximant (*i.e.*, the deviation remains very small (within 0.01 mhartree) for each *a*_x_), denoted as *θ*_3_ (in mhartree):21
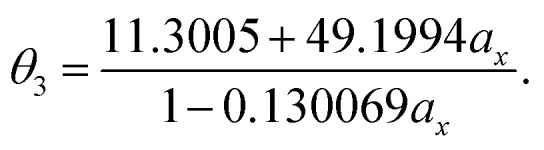


**Fig. 2:**
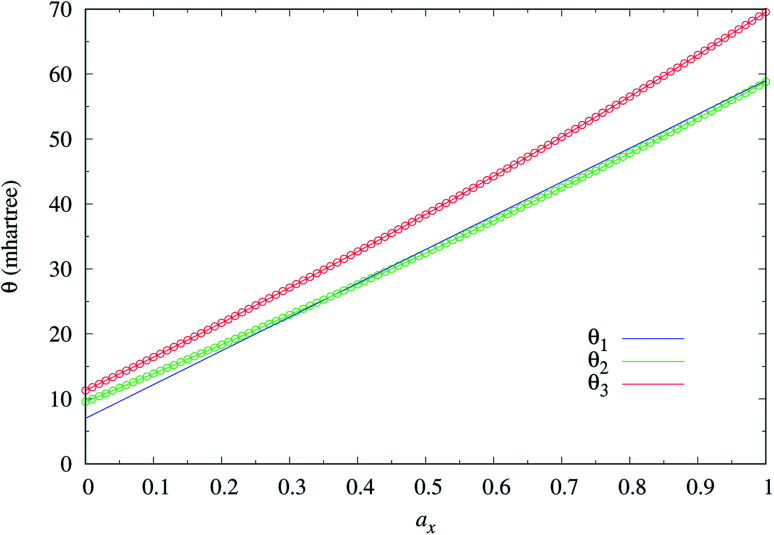
Optimal system-independent fictitious temperature *θ* of TAO-GH (see eqn (13) and (14)) as a function of the fraction of exact exchange *a*_x_. Circles: numerical data of TAO-LDAh (see eqn (16) and (17)) given by the *θ*_A_ (see eqn (11)) and *θ*_B_ (see eqn (12)) models. Lines: analytical parametrizations of *θ*_2_ (see eqn (20)) and *θ*_3_ (see eqn (21)). The analytical parametrization of *θ*_1_ (see eqn (15)) developed in our previous work^32^ is also included for comparison.

As mentioned previously, the underlying DFA functionals in TAO-GH (see eqn (13) and (14)) have insignificant effects on the optimal *θ*. Therefore, while the analytical parametrizations of *θ*_2_ (see eqn (20)) and *θ*_3_ (see eqn (21)) are developed based on the numerical data of TAO-LDAh (see eqn (16) and (17)), the optimal system-independent *θ* of TAO-GH should be reliably given by the parametrizations of *θ*_2_ and *θ*_3_ as well (see Fig. 2). As shown, for each value of *a*_x_, *θ*_2_ remains very close to *θ*_1_ (see eqn (15)), implying that the optimal system-independent *θ* defined in the present work can approximately meet the criterion of optimal system-independent *θ* defined in our previous work.^30–32^ Besides, for each *a*_x_, *θ*_3_ is slightly larger than *θ*_1_ and *θ*_2_. Therefore, for SR systems, it can be anticipated that TAO-GH with the *θ*_1_ or *θ*_2_ parametrization should perform more comparably to the corresponding KS-GH (*i.e.*, TAO-GH with *θ* = 0) than TAO-GH with the *θ*_3_ parametrization.

## IV. Results and discussion

For a comprehensive comparison, we assess the performance of TAO-LDAh with *a*_x_ = 0, 0.25, 0.5, and 1 (denoted as TAO-LDA, TAO-LDAh25, TAO-LDAh50, and TAO-LDAh100, respectively, for brevity) with the corresponding optimal system-independent *θ* values (see Table 1), given by the analytical parametrizations of *θ*_1_ (see eqn (15)), *θ*_2_ (see eqn (20)), and *θ*_3_ (see eqn (21)), on the ground-state properties of several electronic systems with strong static correlation effects, such as the linear acenes (*i.e.*, *n*-acenes) and cyclic carbon chains. The results obtained with the corresponding *θ* = 0 cases, *i.e.*, KS-LDAh with *a*_x_ = 0, 0.25, 0.5, and 1 (denoted as KS-LDA, KS-LDAh25, KS-LDAh50, and KS-LDAh100, respectively, for brevity) are also included for comparison.

**Table 1:** Optimal system-independent fictitious temperature *θ* (in mhartree), given by the analytical parametrizations of *θ*_2_ (see eqn (20)) and *θ*_3_ (see eqn (21)), for TAO-LDA, TAO-LDAh25, TAO-LDAh50, and TAO-LDAh100, where *a*_x_ is the fraction of exact exchange. The corresponding values of *θ*_1_ (see eqn (15)) defined in our previous work^32^ are also included for comparison

	TAO-LDA	TAO-LDAh25	TAO-LDAh50	TAO-LDAh100
*a*_x_	0	0.25	0.5	1
*θ*_1_	7	20	33	59
*θ*_2_	9.55301	20.6214	32.4597	58.7913
*θ*_3_	11.3005	24.3936	38.3974	69.5456

### A. Linear acenes

**Fig. 3:**
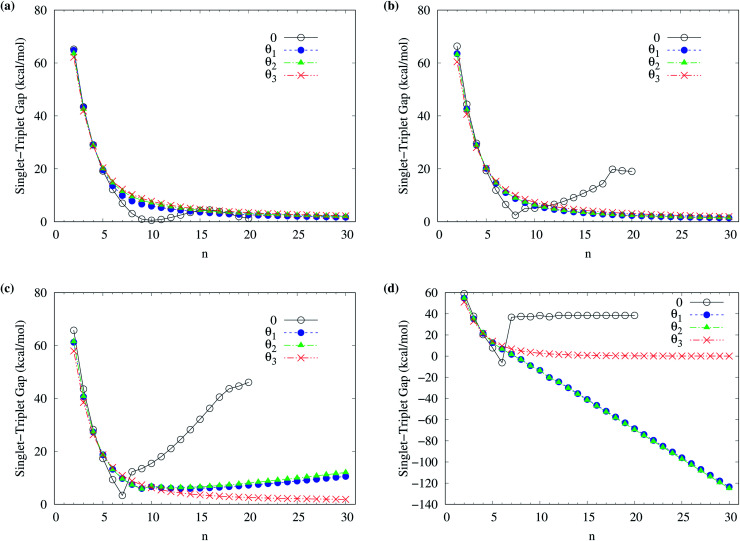
Singlet–triplet gap of *n*-acene (with *n* = 2–30), calculated using spin-unrestricted (a) TAO-LDA, (b) TAO-LDAh25, (c) TAO-LDAh50, and (d) TAO-LDAh100 with the corresponding *θ*_1_, *θ*_2_, and *θ*_3_ values (see Table 1). The *θ* = 0 results are obtained with spin-unrestricted (a) KS-LDA, (b) KS-LDAh25, (c) KS-LDAh50, and (d) KS-LDAh100, respectively.

**Fig. 4:**
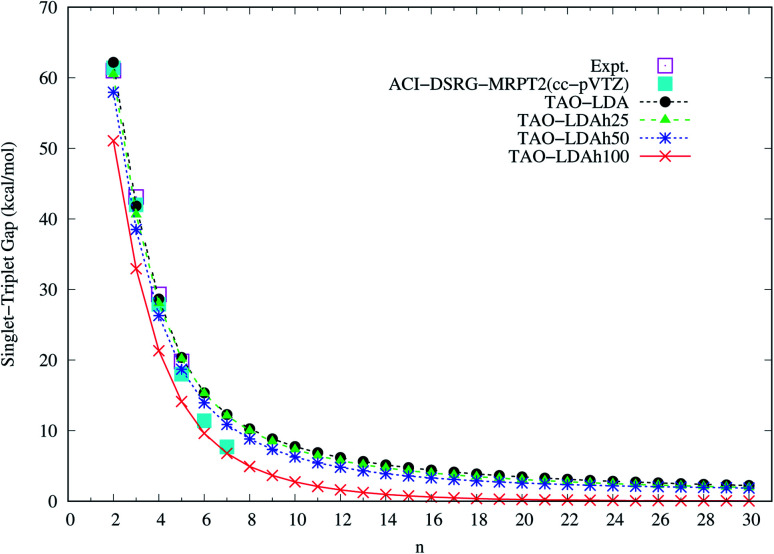
Singlet–triplet gap of *n*-acene (with *n* = 2–30), calculated using spin-unrestricted TAO-LDA, TAO-LDAh25, TAO-LDAh50, and TAO-LDAh100 with the corresponding *θ*_3_ values (see Table 1). The experimental results^57–60^ and the results of ACI-DSRG-MRPT2 (*i.e.*, a reliably accurate MR electronic structure method)^86^ are taken from the literature for comparison.

To determine the ground state of *n*-acene (with *n* = 2–30), we perform spin-unrestricted calculations using TAO-LDA, TAO-LDAh25, TAO-LDAh50, and TAO-LDAh100 with the corresponding *θ*_1_, *θ*_2_, and *θ*_3_ values (see Table 1) for the lowest singlet energy *E*_S_ and lowest triplet energy *E*_T_ of *n*-acene on the respective geometries that were fully optimized at the same level of theory. Subsequently, the singlet–triplet (ST) gap of *n*-acene is computed using *E*_ST_ = *E*_T_ − *E*_S_. The results obtained with the corresponding *θ* = 0 cases (*i.e.*, KS-LDA, KS-LDAh25, KS-LDAh50, and KS-LDAh100, respectively) are also presented for comparison. As shown in Fig. 3, the ST gap of *n*-acene, obtained from TAO-LDA, TAO-LDAh25, TAO-LDAh50 or TAO-LDAh100 with the *θ*_3_ parametrization, decays monotonically with *n*, and remains positive (*i.e.*, a singlet ground state) for each value of *n*, showing consistency with the available experimental results^57–60^ and the results of ACI-DSRG-MRPT2,^86^*i.e.*, a reliably accurate MR electronic structure method (for clarity, also see Fig. 4). Several other theoretical results^64,77,83,84^ have also been in support of the aforementioned behaviors. With the *θ*_3_ parametrization, the ST gaps of *n*-acenes obtained with TAO-LDA, TAO-LDAh25, and TAO-LDAh50 are very similar, which generally agree with the experimental results and the results of ACI-DSRG-MRPT2 for the smaller *n*-acenes (*n* ≤ 5), while for the cases of *n* = 6 and 7, the results of TAO-LDAh100 are closer to the results of ACI-DSRG-MRPT2. Besides, while the results of *θ*_1_ and *θ*_2_ parametrizations are comparable to those of *θ*_3_ parametrization for TAO-LDA and TAO-LDAh25, the results of *θ*_1_ and *θ*_2_ parametrizations can be problematic (*i.e.*, different from the aforementioned behaviors) for TAO-LDAh50 and TAO-LDAh100, especially for the larger *n*-acenes. This implies that for the larger *a*_x_ (*i.e.*, a value between 0.5 and 1), TAO-LDAh with the smaller *θ*_1_ and *θ*_2_ parametrizations can provide insufficient amounts of static correlation for the larger *n*-acenes. On the other hand, the results of *θ* = 0 (*i.e.*, KS-LDA, KS-LDAh25, KS-LDAh50, and KS-LDAh100) all yield unphysical behaviors (*i.e.*, that the ST gap of *n*-acene unexpectedly increases with *n*) for the larger *n*-acenes, indicating that KS-LDAh should be generally inappropriate for studying electronic systems with strong static correlation effects (see Table S1 in ESI†).

For the exact theory, the lowest singlet energy of *n*-acene obtained with a spin-unrestricted calculation must be identical to that obtained with the corresponding spin-restricted calculation because of the spin-symmetry constraint.^4,6,30,75^ To assess whether this constraint can be satisfied, for all the cases, the corresponding spin-restricted calculations are also performed for the lowest singlet energies of *n*-acenes on the respective optimized geometries. The difference between the lowest spin-unrestricted singlet energy *E*_US_ and lowest spin-restricted singlet energy *E*_RS_ of *n*-acene is computed using *E*_UR_ = *E*_RS_ − *E*_US_ (see Fig. S1 in ESI†). As shown, for most cases, KS-LDA, KS-LDAh25, KS-LDAh50, and KS-LDAh100 yield much larger *E*_UR_ values than TAO-LDA, TAO-LDAh25, TAO-LDAh50, and TAO-LDAh100, respectively, with the *θ*_1_, *θ*_2_, and *θ*_3_ parametrizations. In particular, for all the *n*-acenes studied, the *E*_UR_ values obtained from TAO-LDA, TAO-LDAh25, TAO-LDAh50, and TAO-LDAh100 with the *θ*_3_ parametrization are essentially zero (*i.e.*, within the numerical accuracy considered in this work), yielding essentially no unphysical symmetry-breaking effects in the corresponding spin-unrestricted calculations.

Among all the TAO-LDAh calculations, the ground states of *n*-acenes are singlets, except for the *n* ≥ 8 cases of TAO-LDAh100 with the *θ*_1_ and *θ*_2_ parametrizations. For the case where the lowest triplet energy of *n*-acene is lower than the lowest singlet energy of *n*-acene, we also calculate the lowest quintet energy *E*_Q_ of *n*-acene on the respective optimized geometry, and subsequently compute the triplet–quintet (TQ) gap of *n*-acene using *E*_TQ_ = *E*_Q_ − *E*_T_. For the *n* ≥ 8 cases of TAO-LDAh100 with the *θ*_1_ and *θ*_2_ parametrizations, the TQ gaps of *n*-acenes are all positive, and hence the corresponding *n*-acenes have triplet ground states (see Fig. S2 in ESI†).

It can be seen from Fig. 3 and S1† that the results of *θ*_1_ and *θ*_2_ parametrizations are very similar, especially for *a*_x_ ≥ 0.25, just as the values of *θ*_1_ and *θ*_2_ suggest (see Fig. 2 and Table 1). Therefore, hereafter the results of *θ*_2_ parametrization are not presented for brevity. In addition, it can also be seen from Fig. 3 and S1† that the results of *θ* = 0 (*i.e.*, KS-LDA, KS-LDAh25, KS-LDAh50, and KS-LDAh100) all yield unphysical behaviors for the larger *n*-acenes, implying that KS-LDAh should be generally inappropriate for studying MR systems. Accordingly, hereafter the results of *θ* = 0 (*i.e.*, KS-LDA, KS-LDAh25, KS-LDAh50, and KS-LDAh100) are also not included for brevity.

**Fig. 5:**
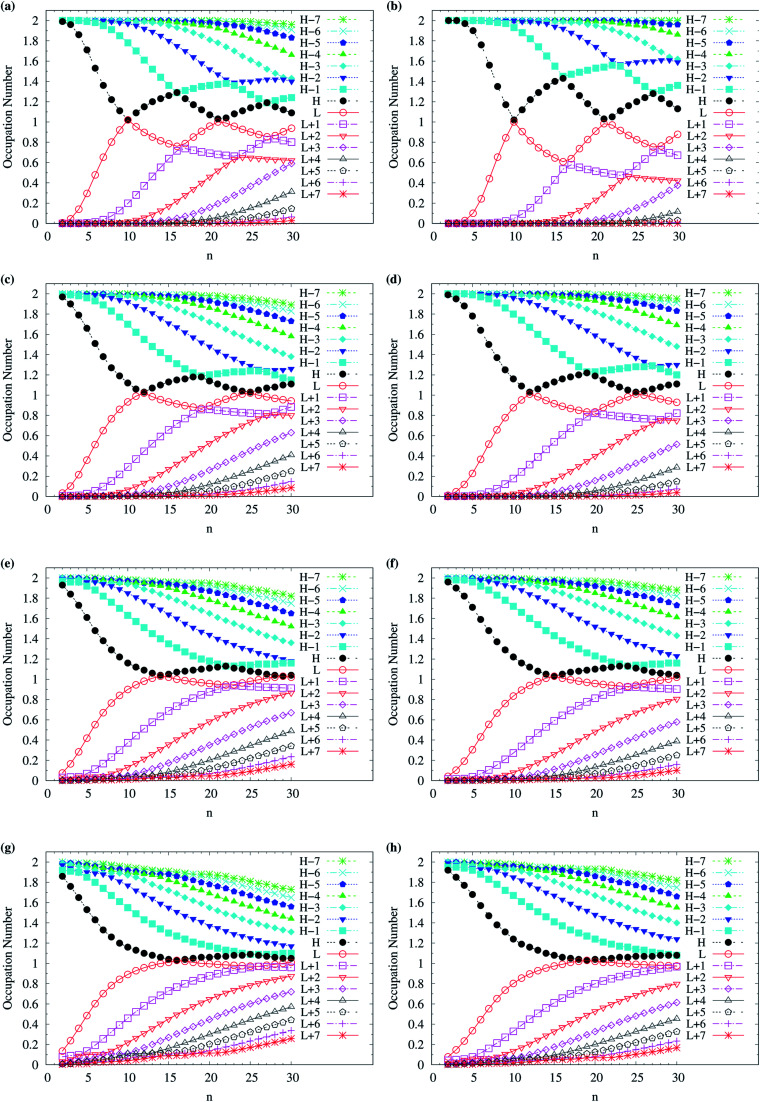
Occupation numbers of active orbitals (HOMO−7, …, HOMO−1, HOMO, LUMO, LUMO+1, …, and LUMO+7) for the lowest singlet state of *n*-acene (with *n* = 2–30), calculated using spin-restricted TAO-LDA with the (a) *θ*_3_ and (b) *θ*_1_ parametrizations, TAO-LDAh25 with the (c) *θ*_3_ and (d) *θ*_1_ parametrizations, TAO-LDAh50 with the (e) *θ*_3_ and (f) *θ*_1_ parametrizations, and TAO-LDAh100 with the (g) *θ*_3_ and (h) *θ*_1_ parametrizations. See Table 1 for the corresponding *θ*_1_ and *θ*_3_ values. Here, HOMO/LUMO is denoted as H/L for brevity.

To assess the possible radical character of *n*-acene, Fig. 5 shows the occupation numbers of active orbitals (HOMO−7, …, HOMO−1, HOMO, LUMO, LUMO+1, …, and LUMO+7) for the lowest singlet state of *n*-acene, calculated using spin-restricted TAO-LDA, TAO-LDAh25, TAO-LDAh50, and TAO-LDAh100 with the *θ*_1_ and *θ*_3_ parametrizations. It can be seen from all the TAO-LDAh results that as *n* increases, the active orbital occupation numbers become closer to 1 (singly occupied) and/or the number of fractionally occupied orbitals (*e.g.*, the orbitals with an occupation number ranging from 0.2 to 1.8) increases, implying that the larger *n*-acenes should possess increasing polyradical character in the lowest singlet states (*i.e.*, showing consistency with the previous results of MR electronic structure methods^64,84^). Moreover, from all the TAO-LDAh results, the active orbital occupation numbers reveal a curve crossing behavior in the approach to 1 (singly occupied) with increasing *n*. For instances, the orbital with the LUMO (HOMO) character in the smaller *n*-acenes can become the HOMO (LUMO) in the larger *n*-acenes. While the curve crossing behavior has been supported by the recent results of an accurate MR electronic structure method,^84^ whether such a curve crossing behavior occurs for the larger *n*-acenes has been under debate.^64,75,77,83–85^

**Fig. 6:**
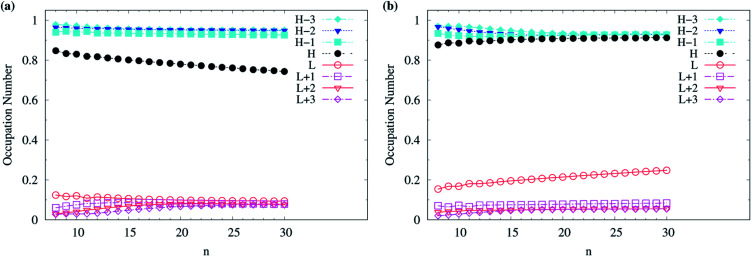
Occupation numbers of (a) α-spin and (b) β-spin active orbitals (HOMO−3, HOMO−2, HOMO−1, HOMO, LUMO, LUMO+1, LUMO+2, and LUMO+3) for the lowest triplet state (*i.e.*, the ground state) of *n*-acene (with *n* = 8–30), calculated using spin-unrestricted TAO-LDAh100 with the corresponding *θ*_1_ value (see Table 1). Here, HOMO/LUMO is denoted as H/L for brevity.

Although various theoretical results^30–32,64,77,83,84,86^ have clearly supported that *n*-acenes should have singlet ground states, for completeness, we also show the occupation numbers of α-spin and β-spin active orbitals (HOMO−3, HOMO−2, HOMO−1, HOMO, LUMO, LUMO+1, LUMO+2, and LUMO+3) for the lowest triplet state of *n*-acene, for the cases where *n*-acenes are predicted to have triplet ground states (*i.e.*, the *n* ≥ 8 cases of TAO-LDAh100 with the *θ*_1_ parametrization). Note that for the lowest triplet state of an electronic system (with *N* electrons), in spin-unrestricted TAO-DFT, the α-spin HOMO is the (*N*/2 + 1)-th α-spin orbital, the α-spin LUMO is the (*N*/2 + 2)-th α-spin orbital, and so on, while the β-spin HOMO is the (*N*/2 − 1)-th β-spin orbital, the β-spin LUMO is the (*N*/2)-th β-spin orbital, and so on. Relative to the active orbital occupation numbers for the lowest singlet state of *n*-acene (see Fig. 5(h)), the occupation numbers of α-spin and β-spin active orbitals for the lowest triplet state of *n*-acene are much closer to either 0 (fully empty) or 1 (fully occupied), and are much less sensitive to the change of acene length (see Fig. 6), implying that the lowest triplet state of *n*-acene, which possesses mainly SR character, can be more accurately described by a SR electronic structure method than the lowest singlet state of *n*-acene.

### B. Cyclic carbon chains

Here, we consider cyclic carbon chains (also called carbon rings), containing *n* carbon atoms in each carbon ring, denoted as c-CC[*n*] (for illustration, see, *e.g.*, Fig. 1(b) of ref. 48) for brevity. Recently, c-CC[*n*] have gained much attention due to their promising properties.^48,93–106^ Among the cyclic carbon chains, c-CC[18] has been recently synthesized.^101^

In our previous study, only TAO-LDA, which contains no exact exchange, has been employed to study the ground-state properties of c-CC[*n*] (with *n* = 10–100).^48^ According to the previous TAO-LDA results, even-numbered c-CC[*n*] should possess increasing polyradical character with the increase of *n*, and should have essentially no bond length alternation (BLA) in their ground states (*i.e.*, the lowest singlet states), contradicting the recent results obtained with the complete-active-space self-consistent-field (CAS-SCF) method (*i.e.*, an accurate MR electronic structure method).^105^ In this work, we argue that these discrepancies are mainly attributed to the severe SIE (also called the delocalization error)^4,6,99^ associated with the LDA XC and *θ*-dependent functional in TAO-DFT (*i.e.*, TAO-LDA).^30–32^ In particular, to minimize the SIE issue, we show that TAO-LDAh100, which contains 100% exact exchange for an improved description of nonlocal exchange effects in TAO-DFT, with the *θ*_1_ and *θ*_3_ parametrizations can successfully resolve the aforementioned discrepancies, yielding the radical character and BLA of even-numbered c-CC[*n*] that are comparable to those obtained with reliably accurate electronic structure methods.

**Fig. 7:**
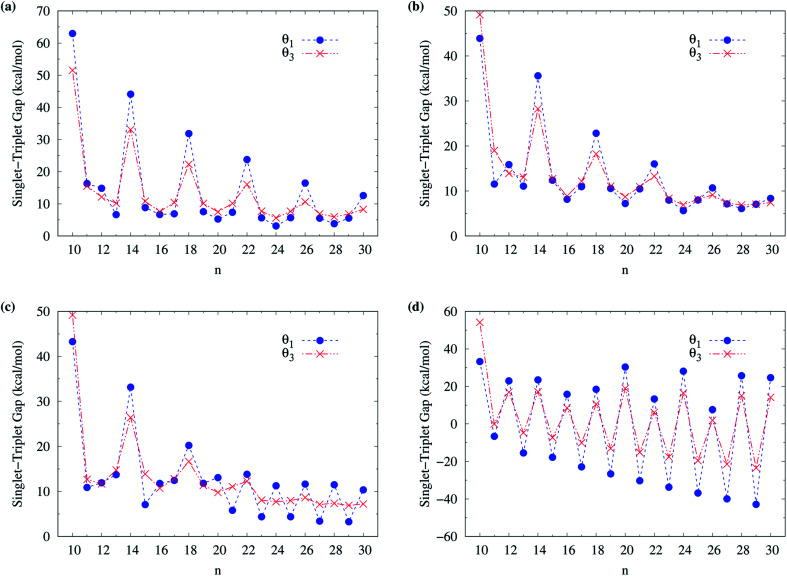
Singlet–triplet gap of c-CC[*n*] (with *n* = 10–30), calculated using spin-unrestricted (a) TAO-LDA, (b) TAO-LDAh25, (c) TAO-LDAh50, and (d) TAO-LDAh100 with the corresponding *θ*_1_ and *θ*_3_ values (see Table 1).

**Fig. 8:**
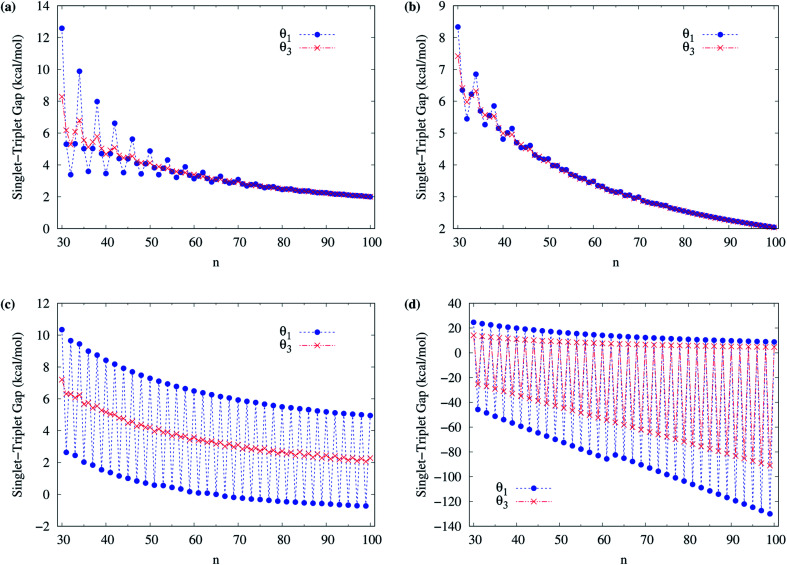
Singlet–triplet gap of c-CC[*n*] (with *n* = 30–100), calculated using spin-unrestricted (a) TAO-LDA, (b) TAO-LDAh25, (c) TAO-LDAh50, and (d) TAO-LDAh100 with the corresponding *θ*_1_ and *θ*_3_ values (see Table 1).

To obtain the ground state of c-CC[*n*] (with *n* = 10–100), we calculate the lowest singlet energy *E*_S_ and lowest triplet energy *E*_T_ of c-CC[*n*] on the respective optimized geometries, using spin-unrestricted TAO-LDA, TAO-LDAh25, TAO-LDAh50, and TAO-LDAh100 with the *θ*_1_ and *θ*_3_ parametrizations (see Table 1), and subsequently, calculate the ST gap (*E*_ST_ = *E*_T_ − *E*_S_) of c-CC[*n*]. As shown in Fig. 7 and 8, the ST gaps of c-CC[*n*] are positive, except for the odd-numbered *n* ≥ 67 cases of TAO-LDAh50 with the *θ*_1_ parametrization and all the odd-*n* cases of TAO-LDAh100 with the *θ*_1_ and *θ*_3_ parametrizations (see Table S2 in ESI†).

As mentioned previously, the difference (*E*_UR_ = *E*_RS_ − *E*_US_) between the lowest spin-unrestricted singlet energy *E*_US_ and lowest spin-restricted singlet energy *E*_RS_ of c-CC[*n*] must be strictly zero for the exact theory due to the spin-symmetry constraint. To examine if this constraint can be obeyed, for all the cases, the corresponding spin-restricted calculations are also performed for the lowest singlet energies of c-CC[*n*] on the respective optimized geometries. For all the c-CC[*n*] studied, the *E*_UR_ values are essentially zero, except for the odd-numbered *n* ≥ 63 cases of TAO-LDAh100 with the *θ*_1_ parametrization (the maximum deviation is within 5.84 kcal mol^−1^) and the odd-numbered *n* ≥ 33 cases of TAO-LDAh100 with the *θ*_3_ parametrization (the maximum deviation is within 0.30 kcal mol^−1^). Note, however, that for these cases (*i.e.*, the cases with noticeable *E*_UR_ values), the ground states of c-CC[*n*] are triplets rather than singlets (as will be shown below).

Among all the TAO-LDAh calculations, the ground states of c-CC[*n*] are singlets, except for the odd-numbered *n* ≥ 67 cases of TAO-LDAh50 with the *θ*_1_ parametrization and all the odd-*n* cases of TAO-LDAh100 with the *θ*_1_ and *θ*_3_ parametrizations. For the case where the lowest triplet energy of c-CC[*n*] is lower than the lowest singlet energy of c-CC[*n*], we also calculate the lowest quintet energy *E*_Q_ of c-CC[*n*] on the respective optimized geometry, and subsequently compute the TQ gap (*E*_TQ_ = *E*_Q_ − *E*_T_) of c-CC[*n*]. For the odd-numbered *n* ≥ 67 cases of TAO-LDAh50 with the *θ*_1_ parametrization and all the odd-*n* cases of TAO-LDAh100 with the *θ*_1_ and *θ*_3_ parametrizations, the TQ gaps of c-CC[*n*] are all positive, and hence the corresponding c-CC[*n*] have triplet ground states (see Fig. S3 in ESI†).

**Fig. 9:**
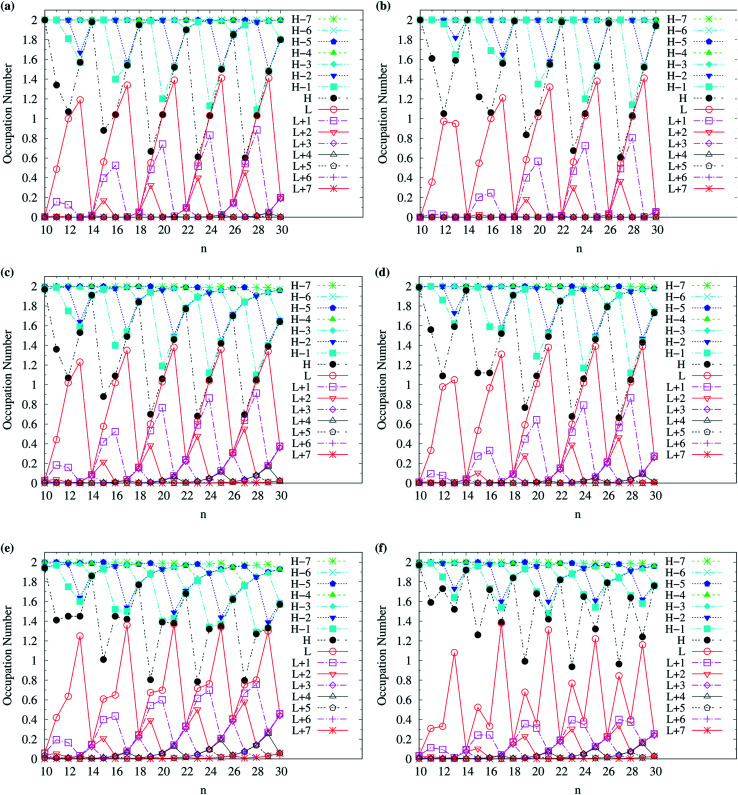
Occupation numbers of active orbitals (HOMO−7, …, HOMO−1, HOMO, LUMO, LUMO+1, …, and LUMO+7) for the lowest singlet state of c-CC[*n*] (with *n* = 10–30), calculated using spin-restricted TAO-LDA with the (a) *θ*_3_ and (b) *θ*_1_ parametrizations, TAO-LDAh25 with the (c) *θ*_3_ and (d) *θ*_1_ parametrizations, and TAO-LDAh50 with the (e) *θ*_3_ and (f) *θ*_1_ parametrizations. See Table 1 for the corresponding *θ*_1_ and *θ*_3_ values. Here, HOMO/LUMO is denoted as H/L for brevity.

**Fig. 10:**
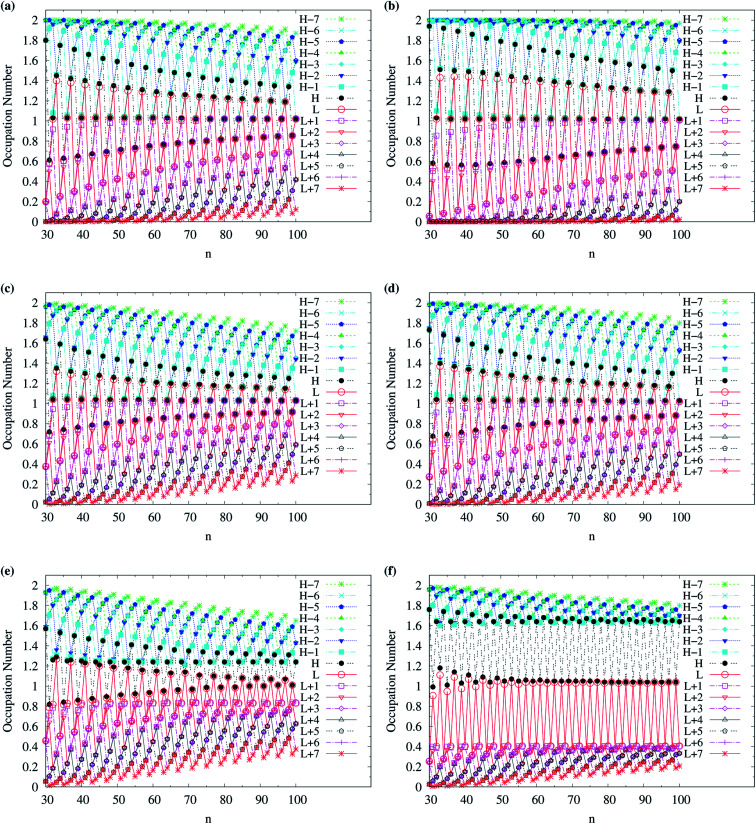
Occupation numbers of active orbitals (HOMO−7, …, HOMO−1, HOMO, LUMO, LUMO+1, …, and LUMO+7) for the lowest singlet state of c-CC[*n*] (with *n* = 30–100), calculated using spin-restricted TAO-LDA with the (a) *θ*_3_ and (b) *θ*_1_ parametrizations, TAO-LDAh25 with the (c) *θ*_3_ and (d) *θ*_1_ parametrizations, and TAO-LDAh50 with the (e) *θ*_3_ and (f) *θ*_1_ parametrizations. See Table 1 for the corresponding *θ*_1_ and *θ*_3_ values. Here, HOMO/LUMO is denoted as H/L for brevity.

To examine the possible radical character of c-CC[*n*], the occupation numbers of active orbitals (HOMO−7, …, HOMO−1, HOMO, LUMO, LUMO+1, …, and LUMO+7) for the lowest singlet state of c-CC[*n*], calculated using spin-restricted TAO-LDA, TAO-LDAh25, and TAO-LDAh50 with the *θ*_1_ and *θ*_3_ parametrizations are shown in Fig. 9 and 10. Similar to our previous findings,^48^ for the cases of TAO-LDA and TAO-LDAh25, the smaller c-CC[4*k* + 2]/c-CC[4*k*] (where *k* are positive integers) generally possess nonradical/tetraradical character, while with the increase of ring size, the active orbital occupation numbers become closer to 1 (singly occupied) and/or the number of fractionally occupied orbitals (*e.g.*, the orbitals with an occupation number ranging from 0.2 to 1.8) increases, suggesting that the larger c-CC[*n*] should possess increasing polyradical character in the lowest singlet states (*i.e.*, the ground states). For the smaller c-CC[*n*], while TAO-LDAh50 with the *θ*_1_/*θ*_3_ parametrization yields a TOON pattern similar to those obtained from TAO-LDA and TAO-LDAh25 with the *θ*_1_/*θ*_3_ parametrization, the degree of tetraradical character of c-CC[4*k*] obtained from TAO-LDAh50 with the *θ*_1_/*θ*_3_ parametrization is apparently much less than that obtained from TAO-LDA and TAO-LDAh25 with the *θ*_1_/*θ*_3_ parametrization. For sufficiently large *n* (*e.g.*, *n* ≥ 30), TAO-LDAh50 with the *θ*_1_/*θ*_3_ parametrization yields a TOON pattern rather different from those obtained from TAO-LDA and TAO-LDAh25 with the *θ*_1_/*θ*_3_ parametrization. Based on the TOON pattern obtained from TAO-LDAh50 with the *θ*_1_ parametrization, even-numbered c-CC[*n*] should possess moderate nonradical character and a low degree of radical character in the lowest singlet states (*i.e.*, the ground states), while odd-numbered c-CC[*n*] (with *n* = 31–65) should possess strong diradical character in the lowest singlet states (*i.e.*, the ground states).

**Fig. 11:**
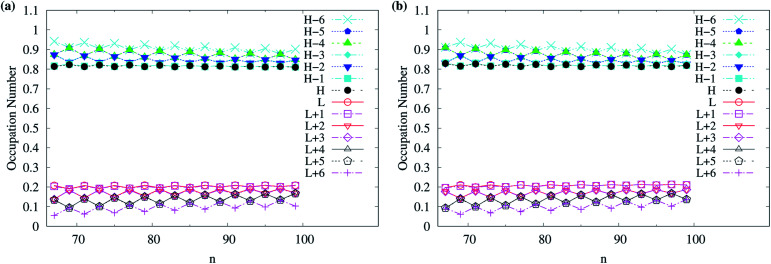
Occupation numbers of (a) α-spin and (b) β-spin active orbitals (HOMO−6, …, HOMO−1, HOMO, LUMO, LUMO+1, …, and LUMO+6) of triplet ground-state odd-numbered c-CC[*n*] (with *n* ≥ 67), calculated using spin-unrestricted TAO-LDAh50 with the corresponding *θ*_1_ value (see Table 1). Here, HOMO/LUMO is denoted as H/L for brevity.

As odd-numbered c-CC[*n*] (with *n* ≥ 67) are predicted to have triplet ground states based on TAO-LDAh50 with the *θ*_1_ parametrization, we also show the occupation numbers of α-spin and β-spin active orbitals (HOMO−6, …, HOMO−1, HOMO, LUMO, LUMO+1, …, and LUMO+6) for the lowest triplet state (*i.e.*, the ground state) of odd-numbered c-CC[*n*] (with *n* ≥ 67), calculated using spin-unrestricted TAO-LDAh50 with the *θ*_1_ parametrization. As shown in Fig. 11, based on TAO-LDAh50 with the *θ*_1_ parametrization, odd-numbered c-CC[*n*] (with *n* ≥ 67) should possess mainly SR character in the lowest triplet states (*i.e.*, the ground states).

**Fig. 12:**
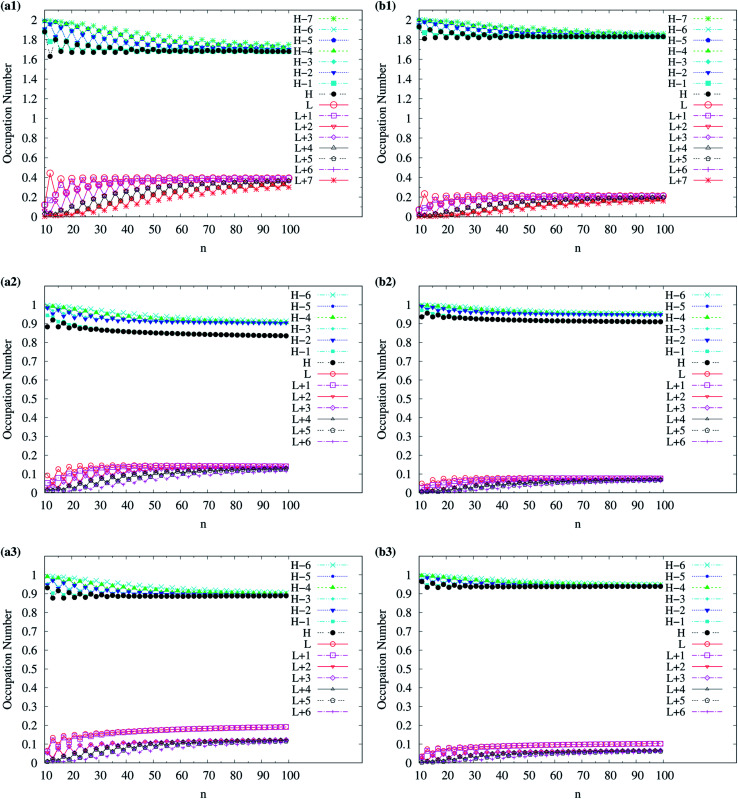
Occupation numbers of active orbitals (HOMO−7, …, HOMO−1, HOMO, LUMO, LUMO+1, …, and LUMO+7) of singlet ground-state even-numbered c-CC[*n*] (with *n* = 10–100), calculated using spin-restricted TAO-LDAh100 with the (a1) *θ*_3_ and (b1) *θ*_1_ parametrizations, the occupation numbers of α-spin active orbitals (HOMO−6, …, HOMO−1, HOMO, LUMO, LUMO+1, …, and LUMO+6) of triplet ground-state odd-numbered c-CC[*n*] (with *n* = 11–99), calculated using spin-unrestricted TAO-LDAh100 with the (a2) *θ*_3_ and (b2) *θ*_1_ parametrizations, and the occupation numbers of β-spin active orbitals (HOMO−6, …, HOMO−1, HOMO, LUMO, LUMO+1, …, and LUMO+6) of triplet ground-state odd-numbered c-CC[*n*] (with *n* = 11–99), calculated using spin-unrestricted TAO-LDAh100 with the (a3) *θ*_3_ and (b3) *θ*_1_ parametrizations. See Table 1 for the corresponding *θ*_1_ and *θ*_3_ values. Here, HOMO/LUMO is denoted as H/L for brevity.

For TAO-LDAh100 with the *θ*_1_ and *θ*_3_ parametrizations, the occupation numbers of active orbitals (HOMO−7, …, HOMO−1, HOMO, LUMO, LUMO+1, …, and LUMO+7) for the lowest singlet state (*i.e.*, the ground state) of even-numbered c-CC[*n*] (with *n* = 10–100), and the occupation numbers of α-spin and β-spin active orbitals (HOMO−6, …, HOMO−1, HOMO, LUMO, LUMO+1, …, and LUMO+6) for the lowest triplet state (*i.e.*, the ground state) of odd-numbered c-CC[*n*] (with *n* = 11–99) are shown in Fig. 12. Based on TAO-LDAh100 with the *θ*_1_/*θ*_3_ parametrization, the (4*k* + 2)/4*k* TOON oscillation pattern is reduced (but still visible) for the smaller even-numbered c-CC[*n*], while with the increase of ring size, these oscillations are gradually reduced, and eventually absent for sufficiently large *n*. Based on the TOON patterns obtained from TAO-LDAh100 with the *θ*_1_/*θ*_3_ parametrization, even-numbered c-CC[*n*] should possess strong/moderate nonradical character (together with only a minimal/low degree of radical character) in the lowest singlet states (*i.e.*, the ground states), showing consistency with the recent results of CAS-SCF,^105^ while odd-numbered c-CC[*n*] should possess mainly SR character in the lowest triplet states (*i.e.*, the ground states).

**Fig. 13:**
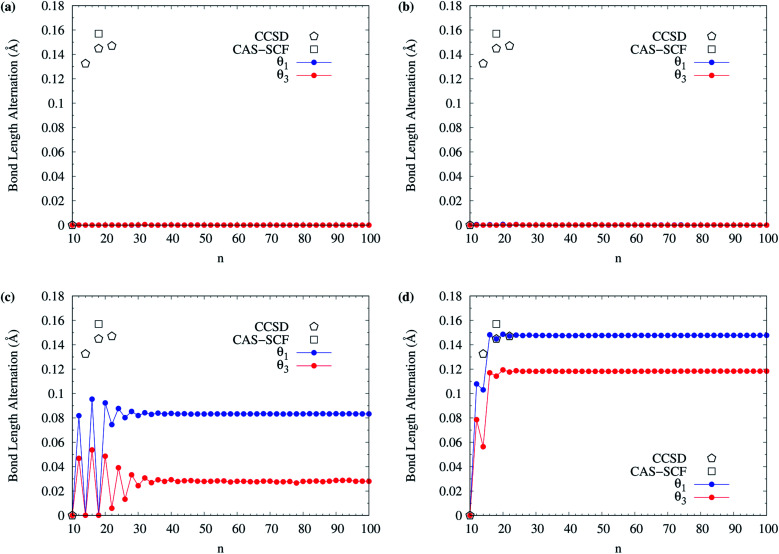
Bond length alternation of singlet ground-state even-numbered c-CC[*n*] (with *n* = 10–100), calculated using spin-restricted (a) TAO-LDA, (b) TAO-LDAh25, (c) TAO-LDAh50, and (d) TAO-LDAh100 with the corresponding *θ*_1_ and *θ*_3_ values (see Table 1). The results of CCSD (with the cc-pVDZ basis set, for *n* = 10, 14, 18, and 22)^97^ and CAS-SCF (for *n* = 18)^100^ are taken from the literature for comparison.

The BLAs of even-numbered c-CC[*n*] have been extensively investigated in recent years.^95–97,99,100,102–106^ As there are two types of bonds (*i.e.*, the alternating long and short bonds) in even-numbered c-CC[*n*], the BLA of even-numbered c-CC[*n*] is defined as the difference between the average long and short bond lengths in even-numbered c-CC[*n*]. In Fig. 13, we show the BLA of singlet ground-state even-numbered c-CC[*n*] (with *n* = 10–100), calculated using spin-restricted TAO-LDA, TAO-LDAh25, TAO-LDAh50, and TAO-LDAh100 with the *θ*_1_ and *θ*_3_ parametrizations (see Table S3 in ESI†). The results obtained from the coupled-cluster theory with iterative singles and doubles (CCSD) using the cc-pVDZ basis set (for *n* = 10, 14, 18, and 22)^97^ and the CAS-SCF method (for *n* = 18)^100^ are taken from the literature for comparison. Similar to our previous findings,^48^ for the cases of TAO-LDA and TAO-LDAh25, the BLAs of even-numbered c-CC[*n*] are vanishingly small (*e.g.*, smaller than 0.001 Å for all the even-*n* cases), yielding the cumulenic structures (*i.e.*, no BLAs) of even-numbered c-CC[*n*]. Due to the severe SIE^4,6,99^ associated with TAO-LDA and TAO-LDAh25,^30–32^ the results of TAO-LDA and TAO-LDAh25 contradict the results of CCSD and CAS-SCF, except only for the *n* = 10 case. Note, however, that the BLAs of even-numbered c-CC[*n*] change drastically with the fraction of exact exchange *a*_x_ adopted in TAO-LDAh. While the BLAs of even-numbered c-CC[*n*], obtained from TAO-LDAh50 with the *θ*_1_ and *θ*_3_ parametrizations remain small (*e.g.*, smaller than 0.10 Å and 0.06 Å, respectively, for all the even-*n* cases), the polyynic structures (*i.e.*, with BLAs) of even-numbered c-CC[*n*] can be correctly obtained for several even-*n* cases (especially for sufficiently large *n*). In particular, the BLAs of even-numbered c-CC[*n*], obtained from TAO-LDAh100 with the *θ*_1_ and *θ*_3_ parametrizations are in good agreement with the results of CCSD and CAS-SCF, correctly yielding the cumulenic structure (*i.e.*, no BLA) of c-CC[10] and the polyynic structures (*i.e.*, with BLAs) of even-numbered c-CC[*n*] (with *n* ≥ 12). On the basis of the TAO-LDAh100 results, for small carbon rings, the BLA of c-CC[4*k* + 2] is smaller than that of the adjacent c-CC[4*k*], which can be explained by the competition between two physical effects: Hückel aromaticity and Jahn–Teller distortion.^96,104^ Besides, as *n* increases, the (4*k* + 2)/4*k* BLA oscillation patterns become more and more indistinguishable, and the BLA of even-numbered c-CC[*n*] approaches a common value (*e.g.*, 0.148 Å and 0.118 Å for TAO-LDAh100 with the parametrizations of *θ*_1_ and *θ*_3_, respectively) for sufficiently large *n* (*e.g.*, *n* ≥ 26), also showing consistency with the recent results of quantum Monte Carlo (QMC) method.^104^

Owing to the much reduced SIE, TAO-LDAh100 with the *θ*_1_/*θ*_3_ parametrization can provide an accurate description of the radical character and BLA of singlet ground-state even-numbered c-CC[*n*], showing consistency with the results of reliably accurate electronic structure methods (*e.g.*, the CCSD,^97^ CAS-SCF,^100,105^ and QMC^104^ methods). Therefore, it can be essential to employ TAO-DFT with exact exchange and an optimal system-independent *θ* (*e.g.*, given by the *θ*_1_, *θ*_2_, or *θ*_3_ parametrization) to accurately predict the ground-state properties of c-CC[*n*] and other electronic systems (where nonlocal exchange effects and strong static correlation effects are important).

## V. Conclusions

In conclusion, we have proposed a simple model to define the optimal system-independent fictitious temperature *θ* of a given energy functional (*i.e.*, a combined XC and *θ*-dependent energy functional) in TAO-DFT.^30–32^ From this model, the optimal system-independent *θ* of an energy functional in TAO-DFT is defined as the corresponding optimal *θ* of an RES (*i.e.*, a boundary between most SR systems and MR systems with diradical character), which is closely related to the HONO occupation number of an RES. On the basis of the arguments presented in this work, 5-acene is chosen as the RES. By taking the HONO occupation number of 5-acene obtained with two reliably accurate MR electronic structure methods, we have developed the *θ*_A_ (see eqn (11)) and *θ*_B_ (see eqn (12)) models to define the optimal system-independent *θ* of an energy functional in TAO-DFT.

In particular, we have employed the *θ*_A_ and *θ*_B_ models to obtain the numerical value of the optimal system-independent *θ* of TAO-LDAh (*i.e.*, a GH functional with the LDA XC and *θ*-dependent energy functionals in TAO-DFT) as a function of the fraction of exact exchange *a*_x_. The numerical data can be accurately represented by the analytical parametrizations of *θ*_2_ (see eqn (20)) and *θ*_3_ (see eqn (21)), respectively, which can be employed to determine the optimal system-independent *θ* of TAO-GH. For each *a*_x_, the *θ* value given by the *θ*_2_ parametrization is very close to that given by the *θ*_1_ (see eqn (15)) parametrization developed previously.^32^ Therefore, the optimal system-independent *θ* defined in this work can approximately meet the criterion of optimal system-independent *θ* defined in our previous work.^30–32^

In addition, we have employed TAO-LDA, TAO-LDAh25, TAO-LDAh50, and TAO-LDAh100 (*i.e.*, TAO-LDAh with *a*_x_ = 0, 0.25, 0.5, and 1, respectively) with the *θ*_1_, *θ*_2_, and *θ*_3_ parametrizations to study the ground-state properties of electronic systems with strong static correlation effects, including the linear acenes (*i.e.*, *n*-acenes) and cyclic carbon chains (*i.e.*, c-CC[*n*]). The results obtained with the corresponding *θ* = 0 cases, *i.e.*, KS-LDA, KS-LDAh25, KS-LDAh50, and KS-LDAh100, respectively, have also been presented for comparison. Besides, the results obtained from experiments and reliably accurate electronic structure methods have been taken from the literature for comparison. For each *a*_x_, while there are small (but noticeable) differences between the TAO-LDAh results obtained with the *θ*_1_ (or the very similar *θ*_2_) and *θ*_3_ parametrizations, TAO-LDAh with the *θ*_1_, *θ*_2_, and *θ*_3_ parametrizations can generally outperform the corresponding KS-LDAh (*i.e.*, TAO-LDAh with *θ* = 0) for MR systems.

Moreover, owing to the much reduced SIE, TAO-LDAh100 with the *θ*_1_ (or the very similar *θ*_2_) and *θ*_3_ parametrizations can accurately predict the radical character and BLA of singlet ground-state even-numbered c-CC[*n*], showing consistency with the results of reliably accurate electronic structure methods. Therefore, for an accurate description of the ground-state properties of electronic systems (where nonlocal exchange effects and strong static correlation effects are important), it can be essential to employ TAO-DFT with exact exchange and an optimal system-independent *θ* (*e.g.*, given by the *θ*_1_, *θ*_2_, or *θ*_3_ parametrization).

## Author contributions

Conceptualization, J.-D. C.; data curation, B.-J. C.; formal analysis, B.-J. C.; funding acquisition, J.-D. C.; investigation, B.-J. C.; methodology, B.-J. C. and J.-D. C.; project administration, J.-D. C.; resources, J.-D. C.; software, J.-D. C.; supervision, J.-D. C.; validation, B.-J. C. and J.-D. C.; visualization, B.-J. C.; writing-original draft, B.-J. C. and J.-D. C.; writing-review & editing, B.-J. C. and J.-D. C. Both authors have read and agreed to the published version of the manuscript.

## Conflicts of interest

There are no conflicts of interest to declare.

## Supplementary Material

RA-012-D2RA01632J-s001
