# Supplementary material for: TAO-DFT fictitious temperature made simple
Source: RSC Adv. 2022 Apr 22;12(19):12193–210. doi: 10.1039/d2ra01632j (PMC9026342; doi:10.1039/d2ra01632j)
Supplement: RA-012-D2RA01632J-s001 [file RA-012-D2RA01632J-s001.pdf]

Electronic Supplementary Material (ESI) for RSC Advances.
This journal is © The Royal Society of Chemistry 2022

Supplementary information to: TAO-DFT fictitious temperature made simple

Bo-Jyun Chen[1] and Jeng-Da Chai[1,2,3,*]

[1]*Department of Physics, National Taiwan University, Taipei 10617, Taiwan*
[2]*Center for Theoretical Physics and Center for Quantum Science and Engineering, National Taiwan University, Taipei 10617, Taiwan*
[3]*Physics Division, National Center for Theoretical Sciences, Taipei 10617, Taiwan*

[*] Author to whom correspondence should be addressed. Electronic mail: jdchai@phys.ntu.edu.tw

[1] J. B. Birks, *Photophysics of Aromatic Molecules*, Wiley, London, 1970.
[2] J. Schiedt and R. Weinkauf, *Chem. Phys. Lett.*, 1997, **266**, 201.
[3] N. Sabbatini, M. T. Indelli, M. T. Gandolfi, and V. Balzani, *J. Phys. Chem.*, 1982, **86**, 3585.
[4] J. Burgos, M. Pope, Ch. E. Swenberg, and R. R. Alfano, *Phys. Status Solidi B*, 1977, **83**, 249.
[5] J. B. Schriber, K. P. Hannon, C. Li, and F. A. Evangelista, *J. Chem. Theory Comput.*, 2018, **14**, 6295–6305.
[6] S. Arulmozhiraja and T. Ohno, *J. Chem. Phys.*, 2008, **128**, 114301.
[7] G. V. Baryshnikov, R. R. Valiev, A. V. Kuklin, D. Sundholm, and H. Ågren, *J. Phys. Chem. Lett.*, 2019, **10**, 6701–6705.

FIGURES

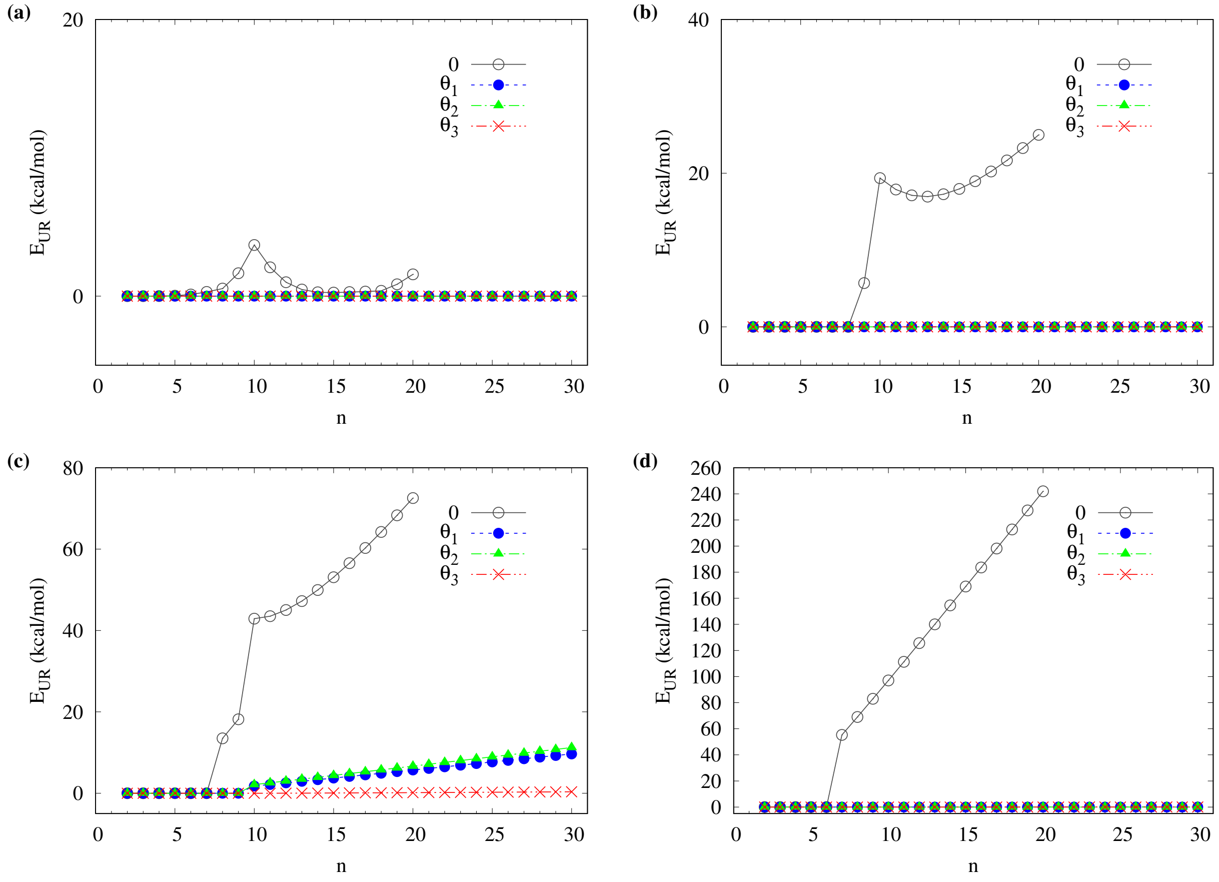

FIG. S1. The difference ($E_{\rm UR} = E_{\rm RS} - E_{\rm US}$) between the lowest spin-unrestricted singlet energy $E_{\rm US}$ and lowest spin-restricted singlet energy $E_{\rm RS}$ of n-acene (with n = 2–30), calculated using (a) TAO-LDA, (b) TAO-LDAh25, (c) TAO-LDAh50, and (d) TAO-LDAh100 with the corresponding θ_1, θ_2, and θ_3 values (see Table I). The $\theta = 0$ results are those obtained with (a) KS-LDA, (b) KS-LDAh25, (c) KS-LDAh50, and (d) KS-LDAh100, respectively.

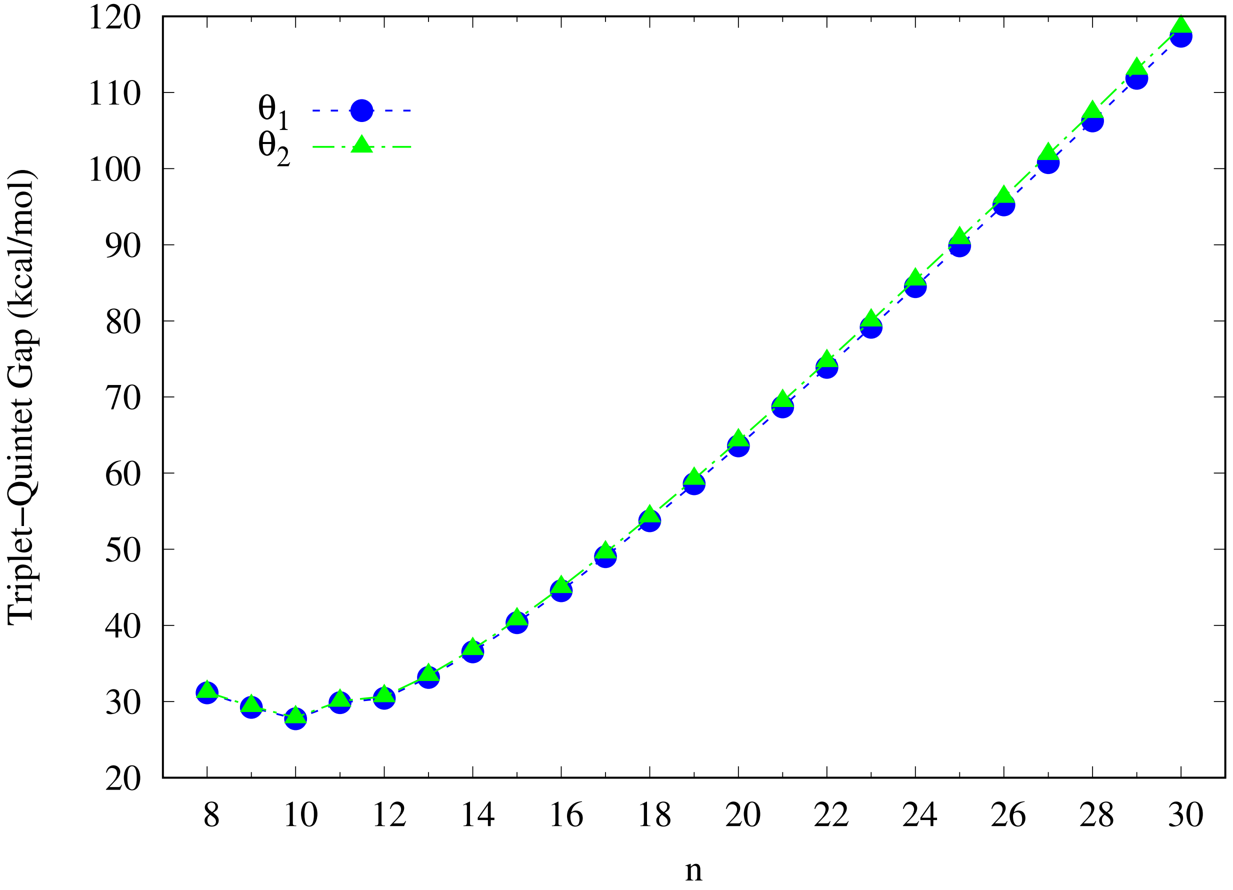

FIG. S2. Triplet-quintet gap of n-acene (with n = 8–30), calculated using spin-unrestricted TAO-LDAh100 with the corresponding θ_1 and θ_2 values (see Table I).

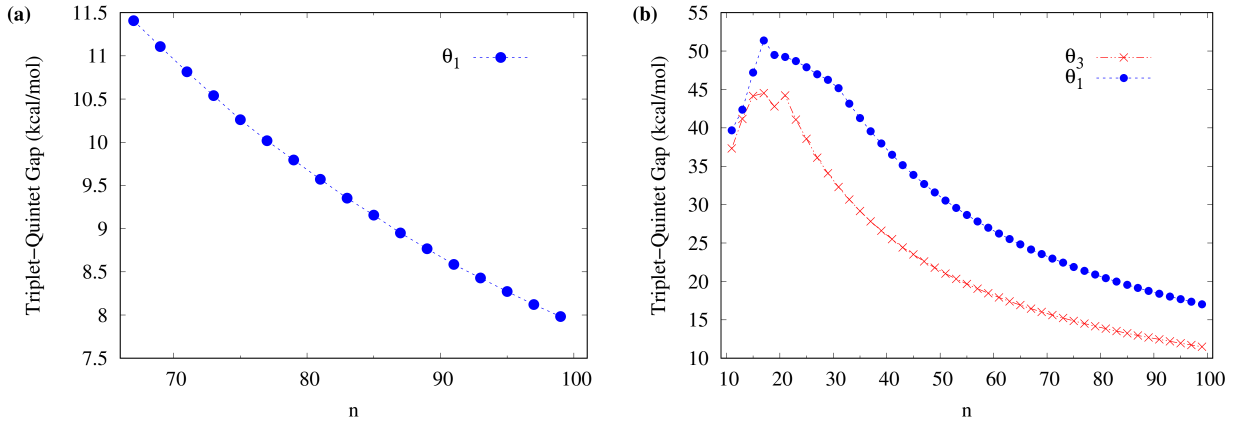

FIG. S3. Triplet-quintet gap of odd-numbered c-CC[n], calculated using spin-unrestricted (a) TAO-LDAh50 with the θ_1 parametrization for $n \geq 67$, and (b) TAO-LDAh100 with the θ_1 and θ_3 parametrizations for $n \geq 11$. See Table I for the corresponding θ_1 and θ_3 values.

TABLES

TABLE S1. Singlet-triplet gap (in kcal/mol) of n-acene, calculated using spin-unrestricted TAO-LDA, TAO-LDAh25, TAO-LDAh50, and TAO-LDAh100 with the corresponding θ_1, θ_2, and θ_3 values (see Table I). The $\theta = 0$ results are obtained with spin-unrestricted KS-LDA, KS-LDAh25, KS-LDAh50, and KS-LDAh100, respectively. The experimental results [1–4] and the results of ACI-DSRG-MRPT2 (denoted as ADMRPT2 for brevity) [5] are taken from the literature for comparison.

n	TAO-LDA				TAO-LDAh25				TAO-LDAh50				TAO-LDAh100				ADMRPT2	Expt.
	θ_3	θ_2	θ_1	$\theta = 0$	θ_3	θ_2	θ_1	$\theta = 0$	θ_3	θ_2	θ_1	$\theta = 0$	θ_3	θ_2	θ_1	$\theta = 0$		
2	62.18	63.55	64.77	65.19	60.45	63.17	63.54	66.34	57.95	61.52	61.24	65.76	51.06	54.81	54.75	59.05	61.4	61.0
3	41.83	42.58	43.22	43.40	40.59	42.33	42.56	44.39	38.50	40.82	40.64	43.54	32.94	35.25	35.22	37.63	42.0	43.1
4	28.64	28.85	29.01	29.06	28.04	28.84	28.95	29.64	26.30	27.38	27.30	28.26	21.31	21.63	21.63	20.38	27.9	29.3
5	20.38	20.09	19.60	19.20	20.19	20.18	20.16	19.34	18.69	18.69	18.71	17.33	14.12	12.74	12.78	7.84	18.0	19.8
6	15.36	14.71	13.55	12.16	15.27	14.69	14.56	11.95	13.93	13.13	13.23	9.32	9.62	6.64	6.73	-6.20	11.4	
7	12.28	11.49	9.91	6.98	12.12	11.21	11.02	6.52	10.87	9.59	9.74	3.34	6.78	1.58	1.73	36.69	7.7	
8	10.26	9.49	7.84	3.07	9.99	8.96	8.74	2.46	8.81	7.32	7.48	12.30	4.91	-3.37	-3.16	37.49		
9	8.83	8.14	6.66	0.99	8.46	7.43	7.21	5.07	7.35	5.79	5.97	13.47	3.65	-9.25	-8.98	37.20		
10	7.74	7.14	5.90	0.53	7.33	6.33	6.12	5.14	6.27	6.89	6.67	15.43	2.75	-13.75	-13.43	38.29		
11	6.88	6.34	5.32	0.86	6.44	5.49	5.30	5.68	5.44	6.51	6.26	18.00	2.08	-20.55	-20.16	37.19		
12	6.19	5.69	4.82	1.59	5.74	4.83	4.65	6.56	4.80	6.36	6.05	21.05	1.61	-24.75	-24.31	38.48		
13	5.62	5.16	4.40	2.52	5.18	4.31	4.14	7.72	4.30	6.35	5.97	24.48	1.23	-30.34	-29.86	38.48		
14	5.15	4.71	3.98	3.50	4.71	3.88	3.72	9.12	3.90	6.43	5.99	28.20	0.97	-35.94	-35.39	38.48		
15	4.75	4.34	3.65	4.34	4.33	3.53	3.37	10.72	3.57	6.60	6.08	32.14	0.75	-41.54	-40.93	38.47		
16	4.42	4.03	3.37	4.43	4.00	3.24	3.09	12.47	3.30	6.82	6.24	36.25	0.59	-47.13	-46.47	38.47		
17	4.12	3.76	3.14	3.90	3.72	2.99	2.85	14.35	3.07	7.08	6.44	40.50	0.47	-52.72	-52.01	38.47		
18	3.87	3.53	2.94	3.01	3.48	2.78	2.65	19.82	2.88	7.38	6.67	43.67	0.38	-58.29	-57.52	38.46		
19	3.64	3.32	2.77	1.96	3.26	2.60	2.47	19.27	2.72	7.71	6.93	44.67	0.29	-63.89	-63.06	38.46		
20	3.44	3.14	2.62	1.30	3.07	2.45	2.32	19.03	2.58	8.06	7.20	46.08	0.25	-69.43	-68.55	38.46		
21	3.26	2.97	2.49		2.91	2.30	2.19		2.46	8.42	7.50		0.19	-74.99	-74.06			
22	3.10	2.83	2.37		2.76	2.18	2.07		2.36	8.79	7.81		0.17	-80.57	-79.57			
23	2.96	2.69	2.25		2.62	2.07	1.96		2.26	9.17	8.13		0.14	-86.13	-85.06			
24	2.82	2.57	2.15		2.50	1.96	1.86		2.19	9.57	8.45		0.12	-91.66	-90.57			
25	2.70	2.46	2.06		2.38	1.88	1.78		2.12	9.96	8.79		0.07	-97.23	-96.04			
26	2.59	2.36	1.98		2.28	1.79	1.70		2.06	10.38	9.13		0.07	-102.72	-101.51			
27	2.49	2.26	1.90		2.19	1.72	1.62		2.00	10.76	9.47		0.07	-108.30	-107.03			
28	2.39	2.18	1.83		2.11	1.64	1.56		1.96	11.19	9.82		0.06	-113.85	-112.52			
29	2.31	2.10	1.76		2.03	1.58	1.50		1.90	11.61	10.18		0.06	-119.36	-117.95			
30	2.22	2.02	1.70		1.95	1.52	1.44		1.86	12.03	10.54		0.04	-124.91	-123.46			

TABLE S2. Singlet-triplet gap (in kcal/mol) of c-CC[n], calculated using spin-unrestricted TAO-LDA, TAO-LDAh25, TAO-LDAh50, and TAO-LDAh100 with the corresponding θ_1 and θ_3 values (see Table I).

n	TAO-LDA		TAO-LDAh25		TAO-LDAh50		TAO-LDAh100	
	θ_3	θ_1	θ_3	θ_1	θ_3	θ_1	θ_3	θ_1
10	51.55	62.97	49.14	43.89	49.19	43.27	54.15	33.24
11	15.52	16.31	18.98	11.54	12.66	10.88	-0.04	-6.62
12	12.13	14.84	13.91	15.86	11.67	11.92	16.92	22.94
13	10.19	6.63	13.06	11.07	14.77	13.72	-4.76	-15.51
14	33.09	44.08	28.25	35.58	26.41	33.16	17.17	23.46
15	10.79	8.82	12.70	12.38	13.88	7.07	-7.34	-17.81
16	7.57	6.67	8.86	8.15	10.73	11.77	8.49	15.81
17	10.40	6.89	12.18	10.96	12.80	12.44	-10.09	-22.91
18	22.28	31.84	18.22	22.82	16.65	20.22	10.51	18.45

19	10.11	7.55	11.05	10.55	11.34	11.80	-13.10	-26.67
20	7.49	5.27	8.71	7.20	9.76	13.07	18.96	30.31
21	10.15	7.36	10.95	10.45	11.05	5.79	-15.17	-30.33
22	16.04	23.75	13.27	16.01	12.25	13.80	6.14	13.31
23	7.71	5.63	8.35	7.96	8.07	4.37	-17.60	-33.75
24	5.60	3.13	6.92	5.64	7.72	11.27	16.45	28.08
25	7.67	5.69	8.16	7.99	7.97	4.36	-19.54	-36.88
26	10.62	16.45	9.11	10.68	8.63	11.63	1.92	7.62
27	6.91	5.52	7.28	7.12	7.11	3.40	-21.49	-39.95
28	5.95	3.81	6.89	6.09	7.36	11.48	14.99	25.70
29	6.84	5.56	7.05	7.04	6.89	3.26	-23.46	-42.90
30	8.28	12.58	7.42	8.33	7.20	10.34	14.14	24.64
31	6.18	5.30	6.41	6.35	6.31	2.64	-25.47	-45.72
32	5.28	3.39	5.98	5.45	6.30	9.65	13.31	23.51
33	6.08	5.33	6.19	6.22	6.07	2.45	-27.12	-48.51
34	6.79	9.88	6.31	6.85	6.24	9.44	12.74	22.59
35	5.56	5.02	5.72	5.69	5.66	2.03	-29.08	-51.25
36	5.12	3.59	5.57	5.26	5.75	8.99	12.13	21.57
37	5.44	5.03	5.51	5.55	5.42	1.84	-31.01	-53.95
38	5.79	7.98	5.53	5.85	5.54	8.75	11.58	20.71
39	5.03	4.71	5.16	5.15	5.28	1.54	-32.96	-56.64
40	4.68	3.46	4.99	4.81	5.17	8.42	11.02	19.92
41	4.92	4.71	4.96	5.01	5.04	1.37	-34.94	-59.30
42	5.08	6.61	4.95	5.14	5.00	8.18	10.57	19.16
43	4.59	4.40	4.70	4.70	4.79	1.15	-36.83	-61.95
44	4.44	3.52	4.64	4.55	4.76	7.91	10.16	18.46
45	4.47	4.38	4.52	4.55	4.47	1.00	-38.81	-64.60
46	4.55	5.62	4.49	4.61	4.57	7.69	9.75	17.78
47	4.22	4.10	4.31	4.31	4.28	0.84	-40.71	-67.25
48	4.13	3.44	4.26	4.22	4.37	7.48	9.39	17.16
49	4.10	4.07	4.15	4.18	4.21	0.71	-42.64	-69.88
50	4.13	4.87	4.12	4.19	4.21	7.29	9.03	16.56
51	3.90	3.82	3.97	3.98	3.95	0.58	-44.57	-72.50
52	3.89	3.39	3.98	3.97	4.11	7.10	8.74	16.03
53	3.78	3.79	3.83	3.86	3.89	0.55	-46.52	-75.13
54	3.79	4.31	3.80	3.85	3.91	6.94	8.41	15.52
55	3.62	3.57	3.69	3.70	3.67	0.43	-48.45	-77.77
56	3.58	3.22	3.66	3.66	3.80	6.78	8.14	15.03
57	3.52	3.53	3.56	3.59	3.61	0.34	-50.40	-80.40
58	3.52	3.88	3.54	3.57	3.65	6.64	7.88	14.57
59	3.38	3.35	3.44	3.45	3.42	0.17	-52.33	-83.01
60	3.39	3.14	3.48	3.48	3.59	6.49	7.64	14.14
61	3.28	3.31	3.33	3.35	3.37	0.09	-54.27	-85.65
62	3.27	3.53	3.30	3.33	3.42	6.38	7.39	13.74
63	3.17	3.15	3.22	3.24	3.26	0.08	-56.21	-82.49
64	3.11	2.93	3.17	3.18	3.34	6.26	7.17	13.35
65	3.08	3.10	3.13	3.14	3.16	0.00	-58.14	-85.11
66	3.10	3.28	3.13	3.16	3.25	6.14	6.98	12.98
67	2.98	2.97	3.03	3.05	3.01	-0.12	-60.07	-87.73
68	2.99	2.87	3.03	3.05	3.17	6.06	6.78	12.63
69	2.90	2.92	2.95	2.96	2.97	-0.19	-62.01	-90.38
70	2.95	3.08	2.97	2.99	3.10	5.93	6.60	12.30

71	2.82	2.81	2.87	2.88	2.85	-0.24	-63.94	-93.03
72	2.77	2.69	2.82	2.83	2.98	5.84	6.41	11.99
73	2.74	2.76	2.78	2.80	2.81	-0.29	-65.88	-95.67
74	2.70	2.80	2.77	2.78	2.90	5.77	6.24	11.69
75	2.67	2.66	2.73	2.74	2.73	-0.32	-67.81	-98.29
76	2.63	2.57	2.71	2.72	2.89	5.66	6.08	11.40
77	2.60	2.61	2.64	2.65	2.65	-0.37	-69.75	-100.94
78	2.57	2.63	2.62	2.62	2.76	5.58	5.93	11.13
79	2.53	2.53	2.58	2.59	2.56	-0.43	-71.68	-103.58
80	2.50	2.46	2.56	2.55	2.71	5.50	5.79	10.87
81	2.47	2.48	2.51	2.52	2.54	-0.47	-73.63	-106.23
82	2.44	2.49	2.48	2.49	2.64	5.44	5.65	10.62
83	2.41	2.41	2.44	2.46	2.44	-0.49	-75.56	-108.87
84	2.38	2.35	2.42	2.43	2.65	5.38	5.53	10.38
85	2.35	2.37	2.39	2.40	2.41	-0.54	-77.51	-111.51
86	2.33	2.36	2.36	2.38	2.56	5.32	5.40	10.15
87	2.30	2.30	2.33	2.35	2.33	-0.56	-79.42	-114.14
88	2.27	2.26	2.31	2.32	2.54	5.24	5.28	9.94
89	2.25	2.26	2.28	2.30	2.29	-0.58	-81.38	-116.81
90	2.22	2.25	2.26	2.27	2.45	5.18	5.16	9.73
91	2.20	2.20	2.23	2.24	2.23	-0.61	-83.30	-119.44
92	2.17	2.16	2.21	2.22	2.41	5.12	5.06	9.53
93	2.15	2.16	2.19	2.20	2.19	-0.65	-85.25	-122.09
94	2.13	2.15	2.16	2.17	2.32	5.08	4.95	9.34
95	2.11	2.11	2.14	2.15	2.15	-0.68	-87.20	-124.74
96	2.08	2.08	2.12	2.13	2.30	5.04	4.85	9.15
97	2.06	2.07	2.10	2.11	2.10	-0.71	-89.13	-127.39
98	2.04	2.06	2.08	2.09	2.24	5.00	4.73	8.97
99	2.02	2.02	2.05	2.06	2.06	-0.73	-91.06	-130.02
100	2.00	2.00	2.03	2.04	2.28	4.95	4.65	8.80

TABLE S3. Bond length alternation (in Å) of singlet ground-state even-numbered c-CC[n], calculated using spin-restricted TAO-LDA, TAO-LDAh25, TAO-LDAh50, and TAO-LDAh100 with the corresponding θ_1 and θ_3 values (see Table I). The results of CCSD (with the cc-pVDZ basis set, for n = 10, 14, 18, and 22) [6] and CAS-SCF (for n = 18) [7] are taken from the literature for comparison.

n	TAO-LDA		TAO-LDAh25		TAO-LDAh50		TAO-LDAh100		CCSD	CAS-SCF
	θ_3	θ_1	θ_3	θ_1	θ_3	θ_1	θ_3	θ_1		
10	0.0000	0.0000	0.0000	0.0000	0.0000	0.0000	0.0000	0.0000	0.0000	
12	0.0001	0.0001	0.0002	0.0005	0.0468	0.0818	0.0785	0.1078		
14	0.0000	0.0000	0.0000	0.0000	0.0001	0.0001	0.0564	0.1030	0.1325	
16	0.0000	0.0000	0.0001	0.0003	0.0537	0.0954	0.1170	0.1482		
18	0.0000	0.0000	0.0000	0.0000	0.0001	0.0002	0.1142	0.1448	0.1448	0.157
20	0.0000	0.0000	0.0001	0.0006	0.0486	0.0923	0.1193	0.1486		
22	0.0001	0.0001	0.0001	0.0000	0.0059	0.0745	0.1176	0.1471	0.1470	
24	0.0000	0.0000	0.0005	0.0006	0.0391	0.0877	0.1187	0.1479		
26	0.0000	0.0000	0.0001	0.0001	0.0133	0.0802	0.1182	0.1475		
28	0.0001	0.0000	0.0001	0.0001	0.0333	0.0853	0.1183	0.1479		
30	0.0001	0.0000	0.0001	0.0001	0.0244	0.0819	0.1182	0.1476		
32	0.0005	0.0005	0.0002	0.0002	0.0307	0.0842	0.1183	0.1476		
34	0.0000	0.0000	0.0001	0.0001	0.0269	0.0829	0.1183	0.1476		

36	0.0000	0.0000	0.0000	0.0000	0.0292	0.0839	0.1182	0.1475
38	0.0000	0.0000	0.0001	0.0001	0.0279	0.0832	0.1182	0.1475
40	0.0002	0.0002	0.0001	0.0001	0.0293	0.0837	0.1182	0.1475
42	0.0000	0.0000	0.0001	0.0001	0.0278	0.0832	0.1182	0.1475
44	0.0000	0.0000	0.0001	0.0001	0.0284	0.0834	0.1182	0.1476
46	0.0001	0.0001	0.0002	0.0002	0.0286	0.0831	0.1182	0.1475
48	0.0001	0.0001	0.0003	0.0003	0.0281	0.0832	0.1182	0.1476
50	0.0000	0.0000	0.0001	0.0001	0.0279	0.0832	0.1182	0.1476
52	0.0000	0.0000	0.0001	0.0000	0.0280	0.0833	0.1182	0.1476
54	0.0000	0.0000	0.0001	0.0001	0.0283	0.0833	0.1182	0.1476
56	0.0000	0.0000	0.0002	0.0002	0.0283	0.0833	0.1183	0.1476
58	0.0000	0.0000	0.0001	0.0001	0.0274	0.0833	0.1182	0.1476
60	0.0000	0.0000	0.0001	0.0001	0.0280	0.0833	0.1182	0.1477
62	0.0000	0.0000	0.0000	0.0000	0.0280	0.0833	0.1182	0.1477
64	0.0001	0.0001	0.0001	0.0001	0.0277	0.0833	0.1182	0.1476
66	0.0000	0.0000	0.0000	0.0000	0.0276	0.0833	0.1184	0.1477
68	0.0000	0.0000	0.0000	0.0001	0.0281	0.0834	0.1183	0.1476
70	0.0001	0.0001	0.0000	0.0000	0.0281	0.0833	0.1182	0.1476
72	0.0000	0.0000	0.0000	0.0001	0.0274	0.0833	0.1182	0.1477
74	0.0000	0.0000	0.0000	0.0002	0.0276	0.0832	0.1183	0.1476
76	0.0000	0.0000	0.0000	0.0000	0.0278	0.0833	0.1183	0.1477
78	0.0000	0.0000	0.0000	0.0000	0.0266	0.0833	0.1183	0.1477
80	0.0000	0.0000	0.0000	0.0000	0.0279	0.0833	0.1183	0.1477
82	0.0000	0.0000	0.0000	0.0000	0.0280	0.0834	0.1183	0.1477
84	0.0000	0.0000	0.0002	0.0002	0.0282	0.0833	0.1183	0.1477
86	0.0000	0.0000	0.0000	0.0000	0.0277	0.0834	0.1184	0.1477
88	0.0000	0.0000	0.0000	0.0000	0.0281	0.0833	0.1183	0.1477
90	0.0000	0.0000	0.0000	0.0000	0.0287	0.0834	0.1183	0.1477
92	0.0001	0.0001	0.0001	0.0001	0.0287	0.0833	0.1184	0.1477
94	0.0000	0.0000	0.0000	0.0000	0.0288	0.0834	0.1183	0.1477
96	0.0000	0.0000	0.0000	0.0000	0.0280	0.0833	0.1183	0.1477
98	0.0000	0.0000	0.0000	0.0000	0.0281	0.0833	0.1184	0.1477
100	0.0000	0.0000	0.0000	0.0000	0.0280	0.0833	0.1184	0.1477
